# Prevalence of co-morbidity and history of recent infection in patients with neuromuscular disease: A cross-sectional analysis of United Kingdom primary care data

**DOI:** 10.1371/journal.pone.0282513

**Published:** 2023-03-01

**Authors:** Iain M. Carey, Niranjanan Nirmalananthan, Tess Harris, Stephen DeWilde, Umar A. R. Chaudhry, Elizabeth Limb, Derek G. Cook

**Affiliations:** 1 Population Health Research Institute, St George’s, University of London, London, United Kingdom; 2 Department of Neurology, St George’s University Hospitals NHS Foundation Trust, London, United Kingdom; Medical College of Wisconsin, UNITED STATES

## Abstract

**Background:**

People with neuromuscular disease (NMD) experience a broader range of chronic diseases and health symptoms compared to the general population. However, no comprehensive analysis has directly quantified this to our knowledge.

**Methods:**

We used a large UK primary care database (Clinical Practice Research Datalink) to compare the prevalence of chronic diseases and other health conditions, including recent infections between 23,876 patients with NMD ever recorded by 2019 compared to 95,295 age-sex-practice matched patients without NMD. Modified Poisson regression estimated Prevalence Ratios (PR) to summarise the presence of the disease/condition ever (or for infections in 2018) in NMD patients versus non-NMD patients.

**Results:**

Patients with NMD had significantly higher rates for 16 of the 18 conditions routinely recorded in the primary care Quality and Outcomes Framework (QOF). Approximately 1-in-10 adults with NMD had ≥4 conditions recorded (PR = 1.39, 95%CI 1.33–1.45). Disparities were more pronounced at younger ages (18–49). For other (non-QOF) health conditions, significantly higher recorded levels were observed for rarer events (pulmonary embolism PR = 1.96 95%CI 1.76–2.18, hip fractures PR = 1.65 95%CI 1.47–1.85) as well as for more common primary care conditions (constipation PR = 1.52 95%CI 1.46–1.57, incontinence PR = 1.52 95%CI 1.44–1.60). The greatest co-morbidity burden was in patients with a myotonic disorder. Approximately 1-in-6 (17.1%) NMD patients had an infection recorded in the preceding year, with the risk of being hospitalised with an infection nearly double (PR = 1.92, 95%CI 1.79–2.07) compared to non-NMD patients.

**Conclusion:**

The burden of chronic co-morbidity among patients with NMD is extremely high compared to the general population, and they are also more likely to present in primary and secondary care for acute events such as infections.

## Introduction

People with neuromuscular disease (NMD) experience a broad range of health issues related to the progression of their disease, such as reduced mobility impacting quality of life [1], as well as pulmonary issues possibly leading to severe respiratory complications [2]. Additional health problems are also associated with specific types of NMD, such as cardiomyopathy in Duchenne or Becker muscular dystrophy [3], endocrine dysfunction in myotonic dystrophy [4] or dysphagia resulting from inflammatory myopathies [5]. Many other associations have been suggested but are less well established, such as a link between myasthenia gravis and diabetes, potentially related to the increased usage of corticosteroids in these patients [6]. Many observations have historically been based on NMD registries [3], and as a result few direct comparisons with the general population exist.

Recent studies have indicated that the combined prevalence of all NMDs may now exceed 100 per 100,000 persons [7], and is rising over time [8]. Previously, we reported on trends in the incidence and prevalence of NMD recorded in UK primary care and showed an increasing burden among older patients [9]. Earlier studies of some NMDs, such as Duchene muscular dystrophy have been based almost exclusively primarily on younger patients, who may be less representative of the overall disease burden in the wider population as the associated life expectancy with the condition has increased over time [10]. As NMD patients are already at a greater risk of falls and fractures from a loss of muscle power over time [1], this risk may become more relevant to an ageing patient group. However, there is an absence of large-scale descriptive studies of older patients with a NMD. Better recognition of older patients with NMD is important, since they are likely to be frequently hospitalised, so better coordinated care might prevent some admissions such as fractures and infections [11].

In this study, we use a large UK primary care database to summarise the chronic diseases, health conditions and recent infections recorded in a group of patients with NMD and compare these directly to a comparator group without NMD, to quantity differences between them. Additionally, we wanted to explore differences by age and type of NMD.

## Material and methods

### Data source

The Clinical Practice Research Datalink (CPRD) is a primary care database in the UK jointly sponsored by the Medicines and Healthcare products Regulatory Agency and the National Institute for Health and Care Research [12]. For over 30 years researchers have used CPRD data to help inform clinical guidance and best practice. Diagnoses are recorded on CPRD using a hierarchical clinical classification system called Read codes [13], from clinical sources such as hospital discharge summaries or communication from specialists. The database recently expanded due to the inclusion of practices using EMIS software [14] and now includes 16 million currently registered patients. Our analysis includes a total of 1,418 practices actively providing data as of 1/1/2019 [9]. Additionally, we also used some data from Hospital Episodes Statistics (HES), which has been linked to CPRD patient records [15]. HES is a data source recording every NHS hospital admission in England, including information on clinical diagnoses [16]. HES linkage was available for 1,088 practices in England in our dataset.

### Classifying patients with neuromuscular disease

Previously, we classified NMD using a hierarchical classification based on the existence of Read codes anywhere previously in their primary care record [9]. As these represent diagnoses made outside of the primary care setting, generally from specialist settings, it is reasonable to assume most diagnoses are valid. Diagnoses were broadly classified into the following (S1 Table): motor neuron disorders, acquired myopathies (e.g. inflammatory myopathies), hereditary myopathies (including muscular dystrophies), mitochondrial disease, muscle channelopathies, hereditary neuropathies (e.g. Charcot-Marie Tooth disease), inflammatory & autoimmune neuropathies (e.g. Guillain-Barré Syndrome), neuromuscular junction disorders (e.g. myasthenia gravis), plus a non-specific category (“Muscular or neuromuscular disease unspecified”) as some Read codes would not allow clear classification into any other category.

For this analysis, we wanted to describe the long-term health in NMD patients, so we excluded patients with motor neurone disease due to the shorter survival time from diagnosis, but still included other motor neuron disorders such as spinal muscular atrophy and post-polio syndrome. We present results for all NMD combined initially, but we also reported findings by the following 6 specific conditions: Charcot-Marie Tooth disease (CMT), Guillain-Barré syndrome (GBS), inflammatory myopathies (IIM), muscular dystrophy (MD), myotonic dystrophy type 1 (DM1) and myasthenia gravis (MG).

### Study cohort and matched non-NMD patients

Patients were included in the study if they were actively registered on 1/1/2019 with their GP and had been so for at least 90 days. We further restricted to NMD patients who had been originally diagnosed at least one year previously. Diagnoses made historically, either at a different practice or pre-computerisation, can be inferred from the record but these will become less reliable the further back in time one goes. Lastly, we only included patients who were at least age 2 on 1/1/2019 as few outcomes in the study would be present below this age. A total of 23,876 patients with NMD were eligible for the analysis (S1 Fig). Four patients matched on age and sex from the same practice without any history of a NMD and registered for >90 days were selected to be the comparator group in the analysis. A total of 95,295 patients without NMD were randomly selected without replacement. Where the outcome required the patient to be registered with their general practice for 1 year (recording of infections), analyses were restricted to 22,946 NMD patients and 87,959 corresponding non-NMD patients who were registered in CPRD throughout 2018. Finally, analyses that relied on linked HES data (England only), were based on 19,012 NMD patients and 74,831 matched non-NMD patients.

### Defining co-morbidity and infections

Our primary focus was describing co-morbidity in NMD patients using a list of conditions routinely collected as part of the Quality and Outcomes Framework (QOF), a UK wide system for performance management and payment of GPs in primary care [17]. Since its introduction in 2004, disease registers for approximately 20 different chronic diseases or conditions have been created and maintained. This has improved data quality and recoding, and we have previously shown how a score based on these conditions is highly predictive of mortality [18]. For the analysis here, we counted the presence of any Read codes for 18 of these conditions (S2 Table) in a patient’s record by 2019, using the published code definitions [17].

Additionally, we also wanted to describe a broader list of health conditions, including many that we would expect to find more commonly in patients with NMD. For this, we created a list of 30 further conditions (S2 Table). The majority of these were selected and adapted from a list of 308 physical and mental health conditions described by Kuan et al [19], who provided a comprehensive summary of recording of prevalence within a subset of CPRD data, including code list definitions. For some conditions we combined some classifications into a broader grouping (cardiomyopathy, uveitis). Finally, we also added constipation and dysphagia to this extended list, due to consensus from primary and secondary care clinician authors on their importance to quality of life in people living with NMD.

For infections, we report only on events recorded in the prior year (2018), additionally utilising the linked HES data to distinguish more serious infections. Infections were grouped into 10 categories: cellulitis, eye, gastro-intestinal, genitourinary, lower respiratory tract, mycoses (candidiasis, other fungal), sepsis, skin and upper respiratory tract. For primary care analyses, presence of an infection for each category was indicated by the occurrence of a Read code in 2018, but we also created a summary group for any infection which also required the prescribing of an antibiotic, antifungal or antiviral drug in the 14 day period either side of the diagnosis, an approach we have used previously [20]. For hospitalisations, any new episode where the primary ICD-10 indicated an infection were counted.

### Statistical analysis

Our summary measure for all analyses was the estimated Prevalence Ratio (PR) to summarise the presence of the condition ever (or for infections in 2018 only) in NMD patients versus non-NMD patients. We used modified Poisson regression, which fits a model with a robust error-variance correction and has been shown to provide reliable relative risk estimates [21]. To account for the matching, a Generalized Estimating Equation (GEE) approach was used which allows for the statistical dependence within the match-sets. All models were fitted using PROC GENMOD in SAS (Version 9.4). Terms for age and sex are included in the model even though they were matched on, but they have little impact on the prevalence ratio due to the balanced design. We also fitted models stratified by age group (e.g., 18–49, 50–64, 65+ for adult comparisons) as it was likely that the prevalence ratio was not constant by age, such that relative comparisons will become less extreme in older ages as the prevalence of disease rises in the general population.

### Ethics approval

This study is based in part on data from the Clinical Practice Research Datalink obtained under licence from the UK Medicines and Healthcare products Regulatory Agency. The protocol (no. 19_211) was approved by the Independent Scientific Advisory Committee evaluation of joint protocols of research involving CPRD data in October 2019. The approval allows analysis of anonymous electronic patient data without the need for written or oral consent.

## Results

### Demographics of cohort

The mean age of the 23,876 patients with NMD included in the study was 54.3 years, with 53.1% of them recorded as being male (Table 1). Among specific neuromuscular disorders, Guillain-Barré syndrome was most common ever recorded (20.1%), followed by Myasthenia Gravis (16.2%) and Charcot-Marie Tooth disease (14.7%). About 23% were not classified any further (“Other”)–these were a combination of rarer conditions (e.g., neuralgic amyotrophy) or non-specific codes (“Myopathy or muscular dystrophy”). A small number of patients (n = 183) were classified into multiple NMD categories and appear in the analysis for each group. Patients with NMD were more likely to consult during 2018 with a GP (S3 Table) and were more likely to have had a GP referral for further care (14.0% vs. 5.8%) than non-NMD patients.

**Table 1:** Demographics of patients in study with a history of neuromuscular disease (NMD) as of 1/1/2019.

	N (%)	Mean age (s.d.)	Median age at diagnosis (IQR)	Number of patients without NMD(%) ‡
**All Patients with NMD** *	23,876	54.3 (20.8)	39 (21–57)	95,295
**Age**				
2 to 17	1,494 (6.3%)	11.1 (4.1)	4 (1–7)	5,967 (6.3%)
18 to 49	7,412 (31.0%)	36.1 (9.0)	22 (12–32)	29,645 (31.1%)
50 to 64	6,298 (26.4%)	57.0 (4.3)	42 (31–50)	25,189 (26.4%)
65+	8,672 (36.3%)	75.3 (7.2)	61 (50–69)	34,494 (36.2%)
**Sex**				
Female	11,206 (46.9%)	55.3 (20.3)	39 (22–56)	44,768 (47.0%)
Male	12,670 (53.1%)	53.4 (21.2)	39 (19–57)	50,527 (53.0%)
**Neuromuscular disorder†**				
Charcot-Marie Tooth	3,511 (14.6%)	51.2 (20.7)	35 (15–53)	14,029 (14.6%)
Guillain-Barré syndrome	4,791 (19.9%)	57.9 (18.4)	40 (24–57)	19,126 (19.9%)
Inflammatory myopathies	2,816 (11.7%)	57.9 (18.6)	45 (28–58)	11,248 (11.7%)
Muscular dystrophy	2,711 (11.3%)	45.3 (22.3)	25 (7–45)	10.832 (11.3%)
Myotonic dystrophy (Type 1)	851 (3.5%)	46.3 (16.6)	30 (18–44)	3,384 (3.5%)
Myasthenia Gravis	3,866 (16.1%)	64.3 (17.2)	55 (35–67)	15,398 (16.0%)
Other	5,519 (22.9%)	50.3 (22.0)	36 (17–53)	22,034 (22.9%)
**Country**				
England	19,735 (82.7%)	54.2 (20.9)	39 (20–57)	78,786 (82.7%)
Northern Ireland	391 (1.6%)	55.4 (19.7)	42 (23–60)	1,563 (1.6%)
Scotland	2,176 (9.1%)	54.1 (20.1)	38 (20–56)	8,676 (9.1%)
Wales	1,574 (6.6%)	55.3 (20.7)	41 (22–57)	6,270 (6.6%)

*—Patients had to be first diagnosed with NMD at least one year previously and required to be at least age 2 as of 1/1/2019.

†—Note that percentages here sum to more than 100% as 183 patients had codes indicating more than 1 NMD.

‡—Patients without NMD were matched on age, sex and GP practice.

Abbreviations: S.D. = standard deviation. IQR = interquartile range.

### Prevalence of chronic disease and health conditions in adults

Among the 18 chronic conditions that were recorded in the QOF (Table 2), 16 of them were significantly higher among patients with NMD (e.g., lower 95% CI for PR was >1). Only serious mental health disorders (e.g., psychosis, schizophrenia, bipolar disorder) and dementia did not show an increased prevalence. The largest relative associations among all NMD patients were seen for learning disability (PR = 2.82), rheumatoid arthritis (PR = 1.94) and osteoporosis (PR = 1.86). Osteoporosis was over 10 times more likely to have been recorded among 18–49-year-olds, as it was extremely rare among non-NMD patients at this age. Similarly in this age group, heart failure (PR = 11.65) and atrial fibrillation (PR = 3.77) produced large prevalence ratios compared to those seen in older age groups. Almost 1-in-4 of NMD patients (24.4%) had ever received a diagnosis of depression, which was 24% higher than the general population and remained constant across age groups. When we summarised multi-morbidity by adding up the total number of QOF conditions ever recorded, approximately 4-in-10 patients with NMD had 2 or more conditions (25% higher than general population), and 1-in-10 had 4 or more (39% higher).

**Table 2:** Prevalence of 18 different chronic conditions recorded in the Quality and Outcomes Framework (QOF) in adults with neuromuscular disease (NMD), and prevalence ratios compared to matched non-NMD patients.

Condition	ALL Adults		Age 18–49		Age 50–64		Age 65-	
	%	PR (95% CI)	%	PR (95% CI)	%	PR (95% CI)	%	PR (95% CI)
Atrial Fibrillation	6.2%	1.28 (1.21,1.36)	0.9%	3.77 (2.69,5.28)	3.1%	1.96 (1.66,2.32)	13.0%	1.17 (1.10,1.24)
Asthma	16.7%	1.21 (1.17,1.25)	19.0%	1.17 (1.11,1.23)	16.0%	1.23 (1.15,1.31)	15.3%	1.25 (1.18,1.32)
Cancer (excl. non-melanoma skin)	8.9%	1.21 (1.15,1.26)	1.9%	1.72 (1.41,2.09)	6.0%	1.22 (1.09,1.37)	17.0%	1.17 (1.11,1.24)
Coronary Heart Disease	8.2%	1.21 (1.15,1.27)	0.6%	1.89 (1.32,2.71)	5.3%	1.42 (1.26,1.61)	16.9%	1.16 (1.10,1.22)
Chronic Kidney Disease	8.1%	1.08 (1.04,1.14)	0.6%	2.39 (1.67,3.42)	3.2%	1.43 (1.22,1.67)	18.1%	1.04 (0.99,1.09)
COPD	4.6%	1.11 (1.04,1.19)	0.5%	1.60 (1.08,2.37)	3.6%	1.21 (1.05,1.40)	8.8%	1.07 (1.00,1.16)
Dementia	1.5%	0.86 (0.77,0.97)	0.0%	2.67 (0.45,15.96)	0.1%	0.85 (0.38,1.92)	3.8%	0.86 (0.77,0.97)
Depression	24.4%	1.24 (1.20,1.27)	22.7%	1.25 (1.19,1.31)	29.4%	1.23 (1.18,1.29)	22.2%	1.23 (1.17,1.28)
Diabetes	13.2%	1.29 (1.24,1.34)	4.1%	1.88 (1.65,2.15)	12.8%	1.37 (1.28,1.48)	21.4%	1.20 (1.14,1.25)
Epilepsy	2.7%	1.72 (1.56,1.88)	3.5%	2.47 (2.12,2.88)	2.5%	1.49 (1.25,1.79)	2.2%	1.32 (1.13,1.56)
Heart Failure	3.2%	1.70 (1.56,1.85)	1.3%	11.65 (7.91,17.14)	1.5%	2.34 (1.82,3.00)	5.9%	1.39 (1.26,1.53)
Hypertension	29.8%	1.09 (1.07,1.12)	5.6%	1.49 (1.34,1.66)	25.4%	1.15 (1.10,1.21)	53.7%	1.05 (1.03,1.08)
Learning Disability	1.3%	2.82 (2.42,3.28)	2.9%	5.36 (4.38,6.56)	0.8%	1.48 (1.08,2.03)	0.2%	0.59 (0.34,1.03)
Mental Health (Psychosis, schizophrenia, bipolar)	1.3%	1.06 (0.93,1.21)	1.3%	1.18 (0.94,1.48)	1.5%	1.08 (0.86,1.36)	1.1%	0.95 (0.76,1.18)
Osteoporosis	6.8%	1.86 (1.76,1.97)	1.7%	10.98 (7.85,15.35)	4.5%	2.86 (2.47,3.31)	12.8%	1.57 (1.48,1.67)
Peripheral arterial disease	1.7%	1.23 (1.10,1.38)	0.1%	2.67 (1.09,6.52)	1.0%	1.74 (1.30,2.32)	3.6%	1.15 (1.02,1.30)
Rheumatoid Arthritis	2.1%	1.94 (1.74,2.16)	0.6%	2.44 (1.70,3.50)	2.1%	2.29 (1.86,2.83)	3.4%	1.76 (1.54,2.01)
Stroke (including TIA)	5.1%	1.31 (1.23,1.40)	0.6%	2.47 (1.72,3.57)	3.3%	1.74 (1.48,2.04)	10.3%	1.21 (1.13,1.30)
2 or more QOF conditions	38.7%	1.25 (1.23,1.27)	14.9%	1.75 (1.64,1.87)	33.3%	1.37 (1.32,1.43)	63.1%	1.14 (1.12,1.16)
4 or more QOF conditions	10.3%	1.39 (1.33,1.45)	0.7%	3.04 (2.14,4.32)	5.8%	2.05 (1.82,2.32)	21.8%	1.29 (1.24,1.35)

**%**—prevalence in NMD patients. **PR**–prevalence ratio and 95% confidence intervals compared non-NMD patients matched on age-sex-practice.

Abbreviations: COPD = Chronic Obstructive Pulmonary Disease, TIA = Transient Ischaemic Attack.

Among the other non-QOF conditions we investigated (S4 Table), the recorded prevalence of rarer conditions such as cardiomyopathy (PR = 4.44), scoliosis (PR = 3.44) and aspiration pneumonitis (3.42) were higher in NMD patients compared to non-NMD patients, as expected. Venous thromboembolism (VTE), either with or without pulmonary embolism, was almost twice as likely to have been recorded, rising to three times higher in those under age 50. More common conditions (constipation, cataract, dysphagia, incontinence, post-viral fatigue syndrome) were all more than 50% higher in NMD patients.

### Prevalence of childhood diseases and conditions

We investigated 12 conditions that were recorded in the children with NMD in our study (S5 Table). Both visual impairment and a history of dysphagia were over 6 times more likely compared to the general population, while sleep apnoea was 4 times more likely. While asthma was higher among adults with NMD, no such association existed among children (PR = 1.00).

### Co-morbidity by type of neuromuscular disease

We repeated the analysis for all different chronic diseases and conditions for the 6 different common NMD groups we investigated. These are summarised in Table 3 and listed according to their relative associations with the general population using the prevalence ratio. The complete set of results for the 18 QOF conditions (S6 Table) and 30 non-QOF (S7 Table) are available in the supplementary material.

**Table 3:** Summary of observed associations in the prevalence of chronic disease and other health conditions in adults with neuromuscular disease (NMD) compared to matched non-NMD patients.

	Charcot-Marie Tooth	Guillain-Barré syndrome	Inflammatory myopathies	Muscular dystrophy	Myotonic dystrophy (Type 1)	Myasthenia Gravis
**>5 times as likely**	Scoliosis	Multiple sclerosis	Aspiration pneumonitis	Cardiomyopathy Scoliosis Aspiration pneumonitis	Cardiomyopathy Aspiration pneumonitis Learning Disability Cataract Sleep apnoea Heart Failure Atrial Fibrillation Visual impairment Dysphagia Autism/Asperger’s Pulmonary embolism	
**>3 times as likely**	Diabetic Neuropathy Sleep apnoea Learning Disability		Rheumatoid Arthritis	Heart Failure Fracture of hip Collapsed vertebra Sleep apnoea Learning Disability	Macular degeneration Scoliosis Skin cancer‡ Diabetic Neuropathy	Aspiration pneumonitis
**>2 times as likely**	Multiple sclerosis Collapsed vertebra Fracture of hip Epilepsy	Diabetic Neuropathy Pulmonary embolism	Cardiomyopathy Dysphagia Sleep apnoea PVFS Pulmonary embolism VTE disease† Osteoporosis Collapsed vertebra	Dysphagia Osteoporosis Diabetic Neuropathy Visual impairment	Collapsed vertebra PAD Constipation Urinary Incontinence VTE disease† IBS	Dysphagia Sleep apnoea Multiple sclerosis Pulmonary embolism
**>50% higher & >10% prevalence**	Constipation Hearing Loss Erectile dysfunction		Hypothyroidism Spondylosis Cataract	Constipation	Hearing Loss	Hypothyroidism Urinary Incontinence
**>20% higher & >20% prevalence**	Osteoarthritis* Depression Anxiety disorders		Erectile dysfunction Osteoarthritis			Depression

*—excludes spine

†—excludes pulmonary embolism

‡—non-melanoma only. Abbreviations: IBS = Irritable Bowel Syndrome, PAD = Peripheral arterial disease, PVFS = Post Viral Fatigue Syndrome, VTE = Venous Thromboembolism.

Patients with CMT not only had a high burden of recorded co-morbidity, but a significantly higher reporting of common conditions impacting quality of life (e.g., constipation, hearing loss, erectile dysfunction, urinary incontinence) than the general population. A history of depression and/or an anxiety disorder was also highest among patients with CMT, with the prevalence higher than the general population (PR = 1.34 depression, PR = 1.21 anxiety). Approximately 1-in-10 CMT patients have received a new depression diagnosis in the last 5 years (n = 355, 10.1%). Diabetic neuropathies were highest in patients with CMT (1.5%, PR = 4.43). To reduce the possibility of misdiagnosis around the same time, we excluded cases with a code for diabetic neuropathy +/- 1 year of their initial CMT diagnosis, but the PR remained high (3.61).

Compared to the other NMDs, patients who have had a prior history of GBS had lower overall co-morbidity for their age, and smaller relative associations for most conditions when compared to their matched non-NMD patients. The anomaly was Multiple Sclerosis (MS) where 1.8% of GBS patients in our study also had a MS diagnosis (PR = 5.59). To try and discount misdiagnosis as explanation, we excluded all match-sets where the GBS case had a first MS diagnosis +/- 1 year of their initial GBS diagnosis, but the PR remained high (4.12). Additionally excluding all GBS cases who had MS at the time of diagnosis in their record still produced an elevated PR (3.16).

Patients with a history of IIM were far more likely to have a range of conditions and complications recorded compared to non-NMD patients, particularly aspiration pneumonia (PR = 8.68) and rheumatoid arthritis (PR = 3.64). Also notable was a history of cancer (PR = 1.44 excluding non-melanoma skin, PR = 1.36 for non-melanoma skin), and diagnoses of coronary heart disease (PR = 1.59) and diabetes (PR = 1.44), which produced higher PRs than for other NMDs.

Patients with myotonic dystrophy type 1 (DM1) had the greatest burden of co-morbidities with 21 different conditions being more than twice as likely to be recorded ever than their matched non-NMD patients. Cardiomyopathy, aspiration pneumonia, learning disability and cataract were all greater than 10 times more likely. An association that appeared specific to DM1 was with non-melanoma skin cancer (PR = 3.31). Diseases of the eye, such as cataracts (PR = 10.93), and circulatory system such atrial fibrillation (PR = 7.59) were particularly raised in DM1 patients compared to other NMDs. Almost 1-in-10 (9.3%) had a co-occurring learning disability, far higher than for any other NMD. While other muscular dystrophies showed a similar pattern for many of these conditions, in general cardiovascular and eye diseases were lower, while musculoskeletal conditions such as hip fractures (PR = 3.92) were higher.

Approximately half (49.8%) of patients with a MG diagnosis had 2 or more QOF conditions. Almost 1-in-4 (23.5%) patients had a diagnosis of diabetes, more the non-NMD group (PR = 1.47). A history of asthma was also noticeably higher in these patients (PR = 1.33). Among other conditions, other raised associations included aspiration pneumonia (PR = 3.45) and dysphagia (PR = 2.92).

### History of recent infection

Table 4 summarises the recording of infections in primary care and hospital admissions for an infection during 2018 among NMD patients (now including children). Among all patients, those with a history of NMD were 43% more likely (PR = 1.43, 95% CI 1.38–1.48) to have had any infection recorded in primary care, affecting 1-in-6 NMD patients (17.1%). A higher risk of infection was seen in all infection categories. When only hospitalisations were counted, the increased risk among NMD patients was now almost a doubling (PR = 1.92, 95%CI 1.79–2.07), with sepsis showing the largest association (PR = 2.37). In both healthcare settings, lower respiratory tract infections were raised among NMD patients, especially among children (PR = 3.63 primary care, PR = 15.1 hospital admissions).

**Table 4:** Occurrence of an infection in the last 12 months in all patients with neuromuscular disease (NMD) compared to matched non-NMD patients.

	ALL Patients		Age 2–17		Age 18–64		Age 65-	
	%	PR (95% CI)	%	PR (95% CI)	%	PR (95% CI)	%	PR (95% CI)
**Recorded in primary care** *								
• Any plus prescription	17.1%	1.43 (1.38,1.48)	17.1%	1.81 (1.57,2.08)	15.1%	1.49 (1.42,1.56)	20.1%	1.33 (1.27,1.40)
• Cellulitis	2.3%	1.65 (1.49,1.82)	0.6%	2.18 (0.92,5.19)	1.4%	1.78 (1.49,2.12)	4.1%	1.57 (1.39,1.78)
• Eye	1.2%	1.37 (1.20,1.57)	1.8%	1.62 (1.03,2.56)	0.9%	1.33 (1.09,1.63)	1.6%	1.36 (1.12,1.65)
• Gastro-Intestinal Tract	0.8%	1.36 (1.16,1.61)	0.7%	1.09 (0.54,2.20)	0.8%	1.51 (1.22,1.88)	0.8%	1.21 (0.92,1.58)
• Genito-Urinary	3.3%	1.46 (1.34,1.58)	0.6%	1.08 (0.52,2.22)	2.6%	1.51 (1.34,1.72)	4.8%	1.41 (1.26,1.58)
• Lower Respiratory Tract	6.8%	1.61 (1.52,1.70)	5.5%	3.63 (2.70,4.87)	5.2%	1.89 (1.72,2.06)	9.4%	1.36 (1.26,1.47)
• Mycoses—Candidiasis	1.2%	1.60 (1.39,1.83)	0.7%	3.17 (1.37,7.33)	1.2%	1.52 (1.27,1.83)	1.3%	1.62 (1.30,2.02)
• Mycoses—Other Fungal	1.7%	1.31 (1.17,1.47)	1.3%	1.28 (0.75,2.18)	1.7%	1.38 (1.18,1.61)	1.8%	1.23 (1.03,1.47)
• Skin (Other)	5.0%	1.37 (1.28,1.46)	5.8%	1.43 (1.12,1.83)	4.9%	1.48 (1.35,1.62)	4.9%	1.22 (1.09,1.35)
• Upper Respiratory Tract (Other)	7.4%	1.23 (1.16,1.29)	15.3%	1.28 (1.11,1.47)	7.8%	1.24 (1.16,1.33)	5.5%	1.18 (1.06,1.30)
**Hospitalisations** **†**								
• Any	5.3%	1.92 (1.79,2.07)	7.5%	5.82 (4.28,7.90)	3.9%	2.47 (2.19,2.78)	7.2%	1.46 (1.32,1.61)
• Gastro-Intestinal Tract	0.9%	2.02 (1.69,2.42)	1.7%	11.90 (5.22,27.12)	0.8%	2.30 (1.77,2.98)	1.0%	1.41 (1.07,1.86)
• Lower Respiratory Tract	2.2%	2.00 (1.78,2.25)	3.5%	15.14 (7.80,29.40)	1.3%	3.61 (2.87,4.53)	3.3%	1.39 (1.19,1.61)
• Sepsis	0.7%	2.37 (1.91,2.94)	0.2%	7.95 (0.72,87.48)	0.5%	3.86 (2.61,5.70)	1.1%	1.86 (1.42,2.43)

**%**—prevalence in NMD patients. **PR**–prevalence ratio and 95% confidence intervals compared to non-NMD patients matched on age-sex-practice.

*—Analysis based on 22,946 NMD patients who were actively registered with their GP throughout 2018 (and 87,959 corresponding age-sex-practice matched non-NMD patients).

†—Analysis based on 19,012 NMD patients who were additionally eligible to be linked to English Hospital Episodes Statistics (HES) data (and corresponding 74,831 age-sex-practice matched non-NMD patients).

When the associations were explored by NMD (S8 Table), the most elevated estimates were seen for Myotonic Dystrophy with patients 80% more likely to have had any infection in primary care (PR = 1.80, 95% CI 1.52–2.13) and any hospitalisation (PR = 2.74, 95%CI 1.85–4.08). This was true across for most infection types except for upper respiratory tract which showed no association. Fungal infections tended to be more common among patients with a history of inflammatory myopathy or myasthenia gravis.

## Discussion

We have used a large UK primary care database to quantify the higher overall burden of recorded chronic diseases, general health conditions and recent infections in NMD patients directly compared to an age-sex-practice matched sample from the general population. To our knowledge, this is a novel comparison and provides a broad overview by age and NMD as to the increased disease burden in these patients.

The main strength of our study is the size, containing over 20,000 patients with recorded NMD from a nationwide sample of general practices in the UK. The CPRD database, at the time of our study date (January 1st 2019), contained approximately 12 million registered patients representing almost 20% of the UK population [9]. So, the results are likely to be generalisable in terms of what is being recorded on primary care medical records across the UK. However, there are several limitations to our analyses.

Firstly, we have not attempted to validate the diagnosis of NMD in our study as we are assuming that any Read codes used for these rare conditions represent diagnoses that have been made in a specialist setting outside of primary care and then transferred to the patients’ GP record [9]. So, while it is possible that some of the patients may have been mis-diagnosed or mis-classified, that would lead to our analysis underestimating the elevated associations we described. An exception here was for GBS and MS where it appears some diagnoses were made closely in time, and the validity of the GBS diagnosis could be queried, as well as acknowledging that historical dates of diagnosis will become less reliable the further back in time they were made. However, the higher finding of MS recorded in patients who have also had GBS still persisted even after excluding these patients.

Secondly, it may be that some of the health conditions we included here are not consistently recorded on GP systems as they are diagnosed outside of primary care (e.g., eye diseases) or they are not always going to result in primary care contact (e.g., constipation). For example, while we found a high prevalence ratio between scoliosis and CMT, only 6.7% of the CMT patients in our study had this recorded, less than the 15% reported in a study of younger CMT patients [22]. A US study of constipation in DMD patients found higher rates than we did, but also reported that less than half were receiving treatment suggesting the condition could be underdiagnosed [23]. Consistency of coding and recording was why we primarily focused on the chronic diseases collated by the QOF. For conditions less consistently recorded, our analysis would be biased if patients with NMD were more likely to be seen and assessed in primary care, which we showed to be the case at all ages during 2018.

Thirdly, an important limitation of our approach is that it is essentially cross-sectional in nature (using a census date of 1^st^ January 2019) and is not exploring the implied future risk of any of these conditions. We have not attempted to disentangle the date ordering of diagnoses and events, and many of the diseases and conditions we reported on would have been recorded before the patient was diagnosed with a NMD, especially as some may be presenting symptoms prior to the initial diagnosis itself. Analyses using CPRD which have explored outcomes post-diagnosis in more common conditions such as chronic inflammatory disorders [24], have shown comparable increases in risk (16%) for depression and anxiety events as we found using our approach here.

Finally, patients with NMD in CPRD who died in 2018 from a complication of their disease are not included in our comparison. So, one might expect that any associations with a health condition associated with short-term mortality such as venous thromboembolism, stroke or sepsis, would be underestimated. For example, while we reported on a large relative association of aspiration pneumonitis ever being recorded in patients for some NMDs, it may still not represent the true risk for this patient group.

Despite these limitations, we think our study provides a broad overview of the overall disease burden encountered by patients with NMD. Among the list of other chronic diseases routinely recorded in UK primary care, almost all were significantly higher with dementia and severe mental illness the only exceptions. Conditions such as depression and diabetes were both relatively common and significantly more likely to have been recorded in patients with NMD. Previous studies have linked CMT to depressive symptoms [25], and MG to diabetes [6]. Since we have previously reported a more than doubling in the prevalence of both recorded CMT and MG in the UK during 2000–19 [9], the number of potential patients with these conditions too will also be increasing. We observed that the recording of diabetic neuropathies was also much higher in patients with NMD, particularly CMT.

An advantage of our analysis was that we were able to stratify comparisons by age and type of NMD, where more meaningful comparisons can be made. In younger adults, the differences are more marked between NMD patents and the general population, where many conditions are rare. Among the different types of NMD we investigated, it was clear that patients with type 1 myotonic dystrophy (DM1) had the greatest disease burden; previous population-based analysis specific to DM1 have demonstrated the level of co-morbidities [26]. In adults with DM1 the frequency of different symptoms has been reported to vary according to age of onset and clinical subtype [27]. Many of the conditions we found with elevated associations with DM1 have been documented in two recent reviews [26,27]. There were two other findings that appeared specific to DM1. We found a higher than expected recording of non-melanoma skin cancer, which mirrors a previous study of DM1 patients using CPRD, that found a higher risk of developing basal cell carcinoma over time [28]. Also of note was the significantly lower prevalence of hypertension compared to non-NMD patients and other NMD, which would back up a historical finding that hypotension was a clinical feature of myotonic dystrophy [29].

Some diseases have not been widely reported in patients with NMD previously. We found higher than expected rates of venous thromboembolism within each NMD, even among the patients with historical GBS diagnoses, many of whom may have recovered over time. The prevalence of DVT in patients with NMD could be presumed to be higher because patients typically have reduced physical activity and may adopt a more sedentary lifestyle [30]. While DVT has been reported as an important cause of mortality in patients with amyotrophic lateral sclerosis and Parkinson disease, there has been limited reporting with other NMD [31]. Although we queried the co-existence of the MS and GBS diagnoses, a case-control study has shown an association between GBS and prior infections such as Epstein-Barr virus [32], which is also thought to be a risk factor for MS [33]; so a more forensic analysis, ideally studied prospectively, is necessary here to understand our finding further.

The associations we observed with osteoporosis and a recorded hip fracture are not surprising given that NMD patients often suffer from nutritional issues impacting bone health, in addition to low levels of physical activity [1]. Falls have also been reported in post-GBS patients, with over half the respondents in a recent survey reporting a fall in the last year [34]. The increase in risk we estimated was quite marked in younger adults compared to the generation population, suggesting that fall prevention methods when developing care plans should be assessed for all adults not just older patients [35]. It has also been advocated that clinicians should consider the administration of anti-osteoporotic medications such as bisphosphonates to prevent fragility fractures due to the prolonged use of glucocorticoids over time [1]. However it is worth noting for MG that neither a previous study using CPRD [36], nor large studies from Canada [37] and Denmark [38] found an increased risk of fracture among MG patients, even when they restricted to those who received high-dose oral glucocorticoids [36,38].

The most novel finding from our analysis, and potentially the most important, may be the consistently higher risk of recent infection in patients in NMD. Since we only included patients with NMD diagnosed prior to 2018, this analysis is based on recorded infections occurring post-diagnosis. So while gastrointestinal and respiratory tract infections have been shown to be associated with an increased risk of developing a IIM [39], our analysis suggests that infection risk may be present both pre- and post-diagnosis for some autoimmune disorders. The higher rates of infection in NMD patients were seen in primary care for common respiratory and skin infections as well as rarer fungal infections. We were able to utilise linked HES data to show that the increased risk for patients with NMD was almost a doubling for hospital admissions for infection, such as sepsis. Many of these admissions are where prevention or effective management in primary care could have decreased the risk of acute hospitalisation [40], so identifying ways to improve surveillance among this group of patients at higher risk could potentially reduce unplanned hospital admissions.

Whilst individually rare, neuromuscular conditions are collectively relatively common with a population prevalence similar to that of Parkinson’s disease or multiple sclerosis [9]. The high levels of medical co-morbidity in patients with neuromuscular conditions highlight the important role of general practitioners in the care of this group of conditions, in terms of recognising and managing treatable co-morbidities and infections which may have a significant effect on quality of life. Case management approaches to linking primary care physicians and community services with specialist neuromuscular services may support care by raising awareness of the spectrum of associated comorbidities in this population and supporting them with early identification of disorder-specific comorbidities.

## Conclusion

We have provided a broad overview of the level of co-morbidity of disease and health conditions experienced by patients with NMD, confirming most well observed associations but also highlighting some less well documented ones, particularly around recent infection.

## Supporting information

S1 Checklist STROBE statement—Checklist of items that should be included in reports of observational studies. (DOC)

S1 Fig Flow chart summarising study design. (DOCX)

S1 Table Classification of neuromuscular disorders used in study analysis. (DOCX)

S2 Table List of health conditions included in analysis. (DOCX)

S3 Table Summary of primary care consultations, referrals, and emergency hospital admissions in 2018 for patients with a neuromuscular disease (NMD) compared to matched non-NMD patients. (DOCX)

S4 Table Prevalence of other (non-QOF) conditions in adults with a neuromuscular disease (NMD), and prevalence ratios compared to matched non-NMD patients. (DOCX)

S5 Table Prevalence of diseases or conditions in children (aged 2–17) with a neuromuscular disease (NMD) and prevalence ratios compared to matched non-NMD patients. (DOCX)

S6 Table Prevalence of 18 different conditions recorded in Quality and Outcomes Framework (QOF) in adults with a neuromuscular disease (NMD), and prevalence ratios compared to matched non-NMD patients, by type of NMD. (DOCX)

S7 Table Prevalence of other non-QOF conditions in adults with a neuromuscular disease (NMD), and prevalence ratios compared to matched non-NMD patients, by type of NMD. (DOCX)

S8 Table Occurrence of an infection in the last 12 months in all patients with a neuromuscular disease (NMD), and prevalence ratios compared to matched non-NMD patients, by type of NMD. (DOCX)
