# Supplementary material for: Prevalence of co-morbidity and history of recent infection in patients with neuromuscular disease: A cross-sectional analysis of United Kingdom primary care data
Source: PLoS One. 2023 Mar 1;18(3):e0282513. doi: 10.1371/journal.pone.0282513 (PMC9977045; doi:10.1371/journal.pone.0282513)
Supplement: S1 Fig — (DOCX) [file pone.0282513.s002.docx]

## **Figure S1:** Flow chart summarising study design

**23,876** patients with a neuromuscular disease (NMD) aged 2+ & registered for >90 days

**95,295** patients (randomly selected) without a NMD registered for >90 days

**January 1^st^, 2019**

N=3,511 Charcot-Marie Tooth

N=4,791 Guillain-Barré syndrome

N=2,816 Inflammatory myopathies

N=2,711 Muscular dystrophy

N=851 Myotonic dystrophy (Type 1)

N=3,866 Myasthenia Gravis

N=5,519 Other NMD

Matched on age, sex and practice

**1,418** general practices providing data to CPRD

**2018**

**22,946** registered for >1 year

**87,959** registered for >1 year, along with corresponding NMD patient

Analysis of recent infection only

**Pre-2018**

Lifetime prevalence of chronic disease
