# Supplementary material for: Prevalence of co-morbidity and history of recent infection in patients with neuromuscular disease: A cross-sectional analysis of United Kingdom primary care data
Source: PLoS One. 2023 Mar 1;18(3):e0282513. doi: 10.1371/journal.pone.0282513 (PMC9977045; doi:10.1371/journal.pone.0282513)
Supplement: S1 Table — (DOCX) [file pone.0282513.s003.docx]

# SUPPLEMENTARY MATERIAL

## **Table S1:** Classification of neuromuscular disease used in study analysis

| Group | Sub-group | Sub-group used in analysis |
| --- | --- | --- |
| Motor Neuron Disorders | Post-Polio syndrome  Spinal muscular atrophy |  |
| Muscle disease | Acquired myopathies  Hereditary myopathies  Mitochondrial disease  Muscle channelopathies  Myotonic disorders (unspecified)^1^ | Inflammatory myopathies  Muscular dystrophies, Myotonic dystrophy (Type 1) |
| Neuropathy | Hereditary Neuropathies Inflammatory & autoimmune neuropathies | Charcot-Marie Tooth disease  Guillain-Barré syndrome |
| Neuromuscular Junction Disorder | Lambert-Eaton syndrome  Myasthenia gravis  Other | Myasthenia gravis |
| Muscular or neuromuscular disease unspecified^2^ |  |  |

1 - This category only applies if they cannot be assigned within “Muscular Dystrophy” or “Muscle channelopathies”
2 - This category only applies if they cannot be assigned to any of the above categories

For Read codes used in the definition please see <https://doi.org/10.24376/rd.sgul.21878271> and also Carey, I. M., et al. (2021). "Prevalence and incidence of neuromuscular conditions in the UK between 2000 and 2019: A retrospective study using primary care data." PloS One 16(12).
