# Supplementary material for: Prevalence of co-morbidity and history of recent infection in patients with neuromuscular disease: A cross-sectional analysis of United Kingdom primary care data
Source: PLoS One. 2023 Mar 1;18(3):e0282513. doi: 10.1371/journal.pone.0282513 (PMC9977045; doi:10.1371/journal.pone.0282513)
Supplement: S2 Table — (DOCX) [file pone.0282513.s004.docx]

## **Table S2:** List of health conditions included in analysis

| Category | Used in Quality and Outcomes Framework (QOF)^1^ | Other conditions^2^ |
| --- | --- | --- |
| Cancer | Cancer (exc. non-melanoma skin) | Non-melanoma skin cancer |
| Circulatory | Atrial Fibrillation | Cardiomyopathy |
|  | Coronary Heart Disease (CHD) | Pulmonary embolism (PE) |
|  | Heart Failure | VTE disease (excluding PE) |
|  | Hypertension |  |
|  | Peripheral arterial disease (PAD) |  |
|  | Stroke (including Transient Ischaemic Attack) |  |
| Digestive |  | Constipation |
|  |  | Dysphagia |
|  |  | Irritable bowel syndrome (IBS) |
| Ear Disease |  | Hearing Loss |
| Endocrine | Chronic Kidney Disease | Hypothyroidism |
|  | Diabetes |  |
| Eye Diseases |  | Cataract |
|  |  | Glaucoma |
|  |  | Macular degeneration |
|  |  | Uveitis |
|  |  | Visual impairment/blindness |
| Genitourinary |  | Erectile dysfunction |
|  |  | Urinary Incontinence |
| Mental Health | Depression | Anxiety disorders |
|  | Learning Disability | Autism/Asperger's syndrome |
|  | Mental Health (including Psychosis, schizophrenia, bipolar) |  |
| Musculoskeletal | Osteoporosis | Collapsed vertebra |
|  | Rheumatoid Arthritis | Fracture of hip |
|  |  | Fracture of wrist |
|  |  | Osteoarthritis (excluding spine) |
|  |  | Scoliosis |
|  |  | Spondylosis |
| Neurological | Dementia | Diabetic Neuropathy |
|  | Epilepsy | Migraine |
|  |  | Multiple sclerosis |
|  |  | Parkinson's disease |
|  |  | Post viral fatigue syndrome (PVFS) |
| Respiratory | Asthma | Aspiration pneumonitis |
|  | Chronic Obstructive Pulmonary Disease (COPD) | Sleep apnoea |

^1^ – Codes lists can be found at the NHS Digital website <https://digital.nhs.uk/data-and-information/data-collections-and-data-sets/data-collections/quality-and-outcomes-framework-qof>

^2^ - Code lists were taken from Kuan et al. (2019), The Lancet Digital Health 1(2): e63-e77 and available at <https://github.com/spiros/chronological-map-phenotypes>
