# Supplementary material for: Prevalence of co-morbidity and history of recent infection in patients with neuromuscular disease: A cross-sectional analysis of United Kingdom primary care data
Source: PLoS One. 2023 Mar 1;18(3):e0282513. doi: 10.1371/journal.pone.0282513 (PMC9977045; doi:10.1371/journal.pone.0282513)
Supplement: S3 Table — (DOCX) [file pone.0282513.s005.docx]

## **Table S3:** Summary of primary care consultations, referrals, and emergency hospital admissions in 2018 for patients with neuromuscular disease (NMD) compared to matched non-NMD patients

|  | NMD Patients | Non-NMD Patients |
| --- | --- | --- |
|  |  |  |
| Total consultations*, Median (IQR) |  |  |
| - Age 2 to 17 | 5 (2-10) | 2 (0-4) |
| - Age 18 to 49 | 5 (2-11) | 3 (0-7) |
| - Age 50 to 64 | 8 (4-16) | 5 (2-10) |
| - Age 65+ | 13 (7-21) | 10 (5-17) |
|  |  |  |
| Has a Read code indicating a referral*, n(%) |  |  |
| - Age 2 to 17 | 199 (14.0%) | 313 (5.8%) |
| - Age 18 to 49 | 1,364 (19.6%) | 2,804 (10.7%) |
| - Age 50 to 64 | 1,499 (24.5%) | 3,516 (14.8%) |
| - Age 65+ | 2,730 (32.3%) | 7,122 (21.8%) |
|  |  |  |
| Has an emergency hospital admission†, n(%) |  |  |
| - Age 2 to 17 | 131 (10.8%) | 174 (3.6%) |
| - Age 18 to 49 | 557 (9.4%) | 1,028 (4.4%) |
| - Age 50 to 64 | 551 (11.2%) | 1,089 (5.6%) |
| - Age 65+ | 1,224 (17.7%) | 3,537 (13.1%) |
|  |  |  |

Note: Non-NMD patients are matched on age-sex-practice

* Primary care summaries restricted to 22,946 NMD patients (n=1,423 age 2-17, n=6,964 age 18-49, n=6,114 age 50-64, n=8,445 age 65+) and corresponding 87,959 matched non-NMD patients (n=5,442 age 2-17, n=26,102 age 18-49, n=23,713 age 50-64, n=32,703 age 65+ who were registered throughout 2018 at their GP practice. Consultation count included prescribing for non-repeat medication and were limited to one consultation per day

† Secondary care summary restricted to 19,012 NMD patients (n=1,212 age 2-17, n=5,957 age 18-49, n=4,940 age 50-64, n=6,903 age 65+) and corresponding 74,831 matched non-NMD patients (n=4,792 age 2-17, n=23,508 age 18-49, n=19,422 age 50-64, n=27,109 age 65+ who were eligible to be linked to English Hospital Episodes Statistics (HES) data
