# Supplementary material for: Prevalence of co-morbidity and history of recent infection in patients with neuromuscular disease: A cross-sectional analysis of United Kingdom primary care data
Source: PLoS One. 2023 Mar 1;18(3):e0282513. doi: 10.1371/journal.pone.0282513 (PMC9977045; doi:10.1371/journal.pone.0282513)
Supplement: S4 Table — (DOCX) [file pone.0282513.s006.docx]

## **Table S4:** Prevalence of other (non-QOF) conditions in adults with neuromuscular disease (NMD), and prevalence ratios compared to matched non-NMD patients

| Condition | ALL Adults | |  | Age 18-49 | | Age 50-64 | | Age 65- | |
| --- | --- | --- | --- | --- | --- | --- | --- | --- | --- |
|  | % | PR (95% CI) |  | % | PR (95% CI) | % | PR (95% CI) | % | PR (95% CI) |
|  |  |  |  |  |  |  |  |  |  |
| Cancer |  |  |  |  |  |  |  |  |  |
| - Non-melanoma skin | 5.9% | 1.21 (1.14,1.28) |  | 0.7% | 1.77 (1.27,2.46) | 3.3% | 1.33 (1.14,1.55) | 12.2% | 1.17 (1.10,1.25) |
| Circulatory System |  |  |  |  |  |  |  |  |  |
| - Cardiomyopathy (any) | 1.6% | 4.44 (3.82,5.18) |  | 2.0% | 21.56 (14.24,32.66) | 1.5% | 5.64 (4.11,7.73) | 1.2% | 1.92 (1.52,2.41) |
| - Pulmonary embolism | 2.2% | 1.96 (1.76,2.18) |  | 0.9% | 3.56 (2.55,4.97) | 1.8% | 2.25 (1.79,2.83) | 3.7% | 1.73 (1.51,1.97) |
| - VTE disease (exc. PE) | 4.6% | 1.72 (1.60,1.85) |  | 2.0% | 2.73 (2.23,3.35) | 3.9% | 2.00 (1.72,2.33) | 7.2% | 1.50 (1.38,1.64) |
| Digestive System |  |  |  |  |  |  |  |  |  |
| - Constipation | 15.4% | 1.52 (1.46,1.57) |  | 12.9% | 1.82 (1.69,1.95) | 12.4% | 1.60 (1.48,1.73) | 19.8% | 1.36 (1.29,1.42) |
| - Dysphagia | 5.2% | 2.21 (2.07,2.37) |  | 3.1% | 3.83 (3.21,4.58) | 5.0% | 2.39 (2.08,2.73) | 7.2% | 1.86 (1.69,2.03) |
| - Irritable bowel syndrome | 9.3% | 1.30 (1.24,1.36) |  | 8.3% | 1.36 (1.25,1.48) | 10.9% | 1.30 (1.20,1.41) | 9.1% | 1.25 (1.16,1.35) |
| Ear Disease |  |  |  |  |  |  |  |  |  |
| - Hearing Loss | 16.1% | 1.27 (1.23,1.31) |  | 8.1% | 1.61 (1.47,1.76) | 11.4% | 1.35 (1.24,1.46) | 26.3% | 1.18 (1.13,1.23) |
| Endocrine |  |  |  |  |  |  |  |  |  |
| - Hypothyroidism | 10.2% | 1.47 (1.41,1.54) |  | 4.9% | 1.72 (1.52,1.93) | 9.9% | 1.53 (1.40,1.67) | 14.9% | 1.39 (1.31,1.47) |
| Eye Diseases |  |  |  |  |  |  |  |  |  |
| - Cataract | 11.8% | 1.50 (1.45,1.56) |  | 2.1% | 7.39 (5.68,9.61) | 6.2% | 2.92 (2.57,3.31) | 24.2% | 1.31 (1.26,1.36) |
| - Glaucoma | 3.2% | 1.29 (1.19,1.40) |  | 0.4% | 2.40 (1.49,3.87) | 1.5% | 1.39 (1.10,1.76) | 6.9% | 1.26 (1.15,1.37) |
| - Macular degeneration | 2.1% | 1.26 (1.14,1.39) |  | 0.1% | 2.29 (0.96,5.45) | 0.5% | 1.63 (1.09,2.43) | 4.9% | 1.23 (1.11,1.36) |
| - Uveitis | 1.3% | 1.24 (1.09,1.41) |  | 0.6% | 1.33 (0.95,1.87) | 1.4% | 1.30 (1.02,1.65) | 2.0% | 1.19 (1.00,1.41) |
| - Visual impairment | 2.2% | 1.87 (1.68,2.08) |  | 1.4% | 3.55 (2.73,4.62) | 1.6% | 2.31 (1.80,2.95) | 3.3% | 1.50 (1.32,1.71) |
| Genitourinary |  |  |  |  |  |  |  |  |  |
| - Erectile dysfunction* | 19.1% | 1.30 (1.24,1.35) |  | 5.9% | 1.68 (1.45,1.95) | 22.5% | 1.50 (1.39,1.62) | 28.1% | 1.16 (1.10,1.22) |
| - Urinary Incontinence | 7.4% | 1.52 (1.44,1.60) |  | 4.0% | 2.08 (1.82,2.39) | 7.5% | 1.55 (1.41,1.72) | 10.2% | 1.37 (1.28,1.47) |
| Mental Health |  |  |  |  |  |  |  |  |  |
| - Anxiety disorders | 20.8% | 1.15 (1.12,1.19) |  | 21.3% | 1.18 (1.13,1.24) | 22.8% | 1.15 (1.09,1.21) | 19.0% | 1.13 (1.07,1.18) |
| - Autism/Asperger's | 0.5% | 2.09 (1.68,2.59) |  | 1.4% | 2.43 (1.93,3.06) | 0.1% | 0.86 (0.40,1.86) | 0.0% | 0.92 (0.26,3.23) |
| Musculoskeletal |  |  |  |  |  |  |  |  |  |
| - Collapsed vertebra | 0.8% | 2.00 (1.68,2.40) |  | 0.2% | 3.33 (1.68,6.61) | 0.3% | 1.63 (0.99,2.68) | 1.6% | 1.99 (1.63,2.43) |
| - Fracture of hip | 1.7% | 1.65 (1.47,1.85) |  | 0.6% | 4.39 (2.88,6.70) | 0.9% | 2.27 (1.65,3.14) | 3.3% | 1.42 (1.25,1.62) |
| - Fracture of wrist | 4.3% | 1.10 (1.03,1.18) |  | 3.3% | 1.03 (0.90,1.18) | 3.4% | 1.15 (0.99,1.34) | 5.7% | 1.12 (1.02,1.23) |
| - Osteoarthritis (exc. spine) | 21.8% | 1.15 (1.12,1.18) |  | 3.3% | 1.55 (1.34,1.79) | 17.8% | 1.24 (1.17,1.31) | 40.7% | 1.11 (1.08,1.15) |
| - Scoliosis | 2.8% | 3.44 (3.09,3.83) |  | 4.7% | 7.39 (6.20,8.79) | 1.6% | 2.62 (2.04,3.35) | 1.9% | 1.78 (1.49,2.14) |
| - Spondylosis | 8.5% | 1.35 (1.29,1.42) |  | 1.0% | 1.52 (1.17,1.97) | 6.1% | 1.40 (1.26,1.57) | 16.7% | 1.34 (1.27,1.41) |
| Neurological |  |  |  |  |  |  |  |  |  |
| - Diabetic Neuropathy | 1.1% | 2.35 (2.00,2.75) |  | 0.2% | 6.18 (2.90,13.20) | 0.9% | 2.47 (1.77,3.46) | 1.9% | 2.18 (1.81,2.62) |
| - Migraine | 9.9% | 1.23 (1.17,1.28) |  | 11.0% | 1.17 (1.09,1.26) | 11.2% | 1.26 (1.16,1.36) | 8.2% | 1.26 (1.17,1.37) |
| - Multiple sclerosis | 0.8% | 2.59 (2.16,3.10) |  | 0.7% | 2.88 (2.00,4.14) | 1.2% | 2.23 (1.67,2.97) | 0.8% | 2.89 (2.12,3.94) |
| - Parkinson's disease | 0.6% | 1.32 (1.09,1.61) |  | 0.0% | 2.00 (0.37,10.92) | 0.2% | 1.44 (0.79,2.61) | 1.3% | 1.31 (1.06,1.62) |
| - Post-viral fatigue synd. | 7.6% | 1.87 (1.77,1.97) |  | 5.9% | 2.15 (1.93,2.40) | 8.8% | 1.80 (1.63,1.98) | 8.1% | 1.78 (1.63,1.94) |
| Respiratory System |  |  |  |  |  |  |  |  |  |
| - Aspiration pneumonitis | 0.5% | 3.42 (2.67,4.38) |  | 0.4% | 6.35 (3.46,11.65) | 0.3% | 3.52 (1.98,6.24) | 0.8% | 2.86 (2.10,3.90) |
| - Sleep apnoea | 3.7% | 2.63 (2.41,2.86) |  | 2.5% | 4.07 (3.32,4.98) | 5.0% | 2.51 (2.19,2.88) | 3.8% | 2.28 (2.00,2.60) |

**%** - prevalence in NMD patients. **PR** – prevalence ratio and 95%CI compared to non-NMD patients matched on age-sex-practice, * - men only
