# Supplementary material for: Prevalence of co-morbidity and history of recent infection in patients with neuromuscular disease: A cross-sectional analysis of United Kingdom primary care data
Source: PLoS One. 2023 Mar 1;18(3):e0282513. doi: 10.1371/journal.pone.0282513 (PMC9977045; doi:10.1371/journal.pone.0282513)
Supplement: S5 Table — (DOCX) [file pone.0282513.s007.docx]

## **Table S5:** Prevalence of diseases or conditions in children (aged 2-17) with neuromuscular disease (NMD) and prevalence ratios compared to matched non-NMD patients

|  | All Children | |
| --- | --- | --- |
|  | % | PR (95% CI) |
|  |  |  |
| Digestive System |  |  |
| - Constipation | 23.0% | 1.46 (1.31,1.63) |
| - Dysphagia | 1.7% | 6.24 (3.39,11.51) |
| Ear Disease |  |  |
| - Hearing Loss | 7.1% | 1.71 (1.37,2.12) |
| Eye Diseases |  |  |
| - Visual impairment | 1.7% | 6.24 (3.23,12.07) |
| Genitourinary |  |  |
| - Urinary Incontinence | 1.5% | 1.41 (0.88,2.27) |
| Mental Health |  |  |
| - Anxiety disorders | 3.8% | 1.58 (1.17,2.14) |
| - Autism/Asperger's | 5.5% | 1.82 (1.41,2.34) |
| - Learning Disability | 1.4% | 3.65 (2.02,6.59) |
| Neurological |  |  |
| - Migraine | 1.9% | 1.32 (0.86,2.01) |
| Respiratory System |  |  |
| - Aspiration pneumonitis | 0.9% | N/A |
| - Asthma | 10.6% | 1.00 (0.86,1.18) |
| - Sleep apnoea | 2.1% | 3.99 (2.45,6.52) |

**%** - prevalence in NMD patients. **PR** – prevalence ratio and 95%CI compared to non-NMD patients matched on age-sex-practice. **N/A** – Not available as there were no occurrences in the non-NMD patients
