# Supplementary material for: Prevalence of co-morbidity and history of recent infection in patients with neuromuscular disease: A cross-sectional analysis of United Kingdom primary care data
Source: PLoS One. 2023 Mar 1;18(3):e0282513. doi: 10.1371/journal.pone.0282513 (PMC9977045; doi:10.1371/journal.pone.0282513)
Supplement: S6 Table — (DOCX) [file pone.0282513.s008.docx]

## **Table S6:** Prevalence of 18 different conditions recorded in Quality and Outcomes Framework (QOF) in adults with neuromuscular disease (NMD), and prevalence ratios compared to matched non-NMD patients, by type of NMD

| Condition | Charcot-Marie Tooth | | Guillain-Barré syndrome | | Inflammatory myopathies | | Muscular dystrophy | | Myotonic dystrophy (T1) | | Myasthenia Gravis | |
| --- | --- | --- | --- | --- | --- | --- | --- | --- | --- | --- | --- | --- |
|  | % | PR (95% CI) | % | PR (95% CI) | % | PR (95% CI) | % | PR (95% CI) | % | PR (95% CI) | % | PR (95% CI) |
| Atrial Fibrillation | 5.0% | 1.26 (1.08,1.48) | 5.9% | 1.15 (1.01,1.30) | 6.2% | 1.39 (1.18,1.63) | 4.6% | 1.27 (1.03,1.57) | 11.6% | 7.59 (5.36,10.74) | 8.1% | 1.03 (0.92,1.15) |
| Asthma | 17.2% | 1.20 (1.10,1.30) | 16.1% | 1.22 (1.13,1.31) | 15.5% | 1.14 (1.03,1.26) | 14.6% | 1.01 (0.90,1.12) | 14.9% | 0.96 (0.80,1.15) | 17.4% | 1.33 (1.23,1.44) |
| Cancer* | 6.6% | 1.03 (0.90,1.19) | 9.1% | 1.22 (1.10,1.35) | 11.9% | 1.44 (1.28,1.61) | 5.8% | 1.05 (0.88,1.26) | 4.8% | 1.07 (0.75,1.53) | 13.0% | 1.26 (1.15,1.39) |
| Coronary Heart Disease | 6.5% | 1.13 (0.98,1.29) | 8.0% | 1.10 (0.99,1.22) | 9.9% | 1.59 (1.39,1.80) | 7.0% | 1.35 (1.15,1.59) | 3.6% | 1.63 (1.10,2.40) | 11.2% | 1.11 (1.01,1.23) |
| Chronic Kidney Disease | 5.0% | 0.80 (0.69,0.93) | 8.1% | 1.06 (0.96,1.18) | 10.0% | 1.20 (1.07,1.35) | 4.4% | 0.95 (0.78,1.14) | 3.6% | 1.37 (0.95,1.97) | 13.1% | 1.13 (1.04,1.23) |
| COPD | 4.1% | 1.09 (0.90,1.30) | 4.1% | 1.01 (0.87,1.18) | 4.9% | 1.11 (0.93,1.34) | 3.2% | 0.99 (0.78,1.27) | 3.0% | 1.37 (0.87,2.18) | 5.9% | 1.05 (0.91,1.21) |
| Dementia | 1.2% | 0.89 (0.64,1.23) | 1.4% | 0.76 (0.59,0.98) | 1.9% | 1.07 (0.80,1.44) | 0.9% | 0.91 (0.58,1.43) | 0.6% | 1.32 (0.41,4.26) | 2.4% | 0.81 (0.65,1.00) |
| Depression | 26.2% | 1.34 (1.26,1.43) | 23.3% | 1.19 (1.12,1.26) | 25.3% | 1.19 (1.11,1.28) | 22.7% | 1.16 (1.07,1.26) | 24.8% | 1.11 (0.97,1.27) | 23.5% | 1.20 (1.13,1.28) |
| Diabetes | 10.5% | 1.15 (1.03,1.28) | 12.1% | 1.11 (1.02,1.21) | 15.5% | 1.44 (1.31,1.59) | 10.2% | 1.24 (1.08,1.41) | 8.1% | 1.31 (1.01,1.69) | 19.1% | 1.47 (1.37,1.59) |
| Epilepsy | 2.8% | 2.11 (1.65,2.71) | 1.9% | 1.11 (0.88,1.39) | 1.8% | 1.16 (0.85,1.58) | 2.9% | 1.73 (1.31,2.29) | 2.5% | 1.65 (0.96,2.84) | 2.9% | 1.67 (1.34,2.07) |
| Heart Failure | 2.0% | 1.33 (1.02,1.73) | 2.2% | 1.21 (0.98,1.49) | 3.4% | 1.88 (1.48,2.38) | 5.9% | 3.99 (3.17,5.02) | 4.8% | 9.60 (5.48,16.82) | 4.0% | 1.31 (1.10,1.57) |
| Hypertension | 24.5% | 1.04 (0.98,1.10) | 30.8% | 1.08 (1.03,1.13) | 34.7% | 1.16 (1.11,1.23) | 24.8% | 1.19 (1.11,1.28) | 9.8% | 0.66 (0.53,0.81) | 39.8% | 1.10 (1.05,1.14) |
| Learning Disability | 1.3% | 3.07 (2.06,4.57) | 0.4% | 0.94 (0.58,1.53) | 0.5% | 1.04 (0.56,1.91) | 1.9% | 3.24 (2.21,4.76) | 9.3% | 13.59 (8.42,21.94) | 0.4% | 1.28 (0.71,2.28) |
| Mental Health | 1.1% | 0.99 (0.69,1.40) | 1.3% | 1.07 (0.81,1.40) | 1.2% | 0.99 (0.68,1.43) | 1.2% | 1.18 (0.78,1.81) | 1.0% | 0.65 (0.29,1.48) | 1.2% | 0.99 (0.72,1.37) |
| Osteoporosis | 4.8% | 1.82 (1.53,2.17) | 5.2% | 1.37 (1.20,1.57) | 11.0% | 2.02 (1.78,2.30) | 6.9% | 2.65 (2.19,3.21) | 2.2% | 1.43 (0.85,2.41) | 9.8% | 1.83 (1.63,2.04) |
| Peripheral arterial disease | 1.5% | 1.23 (0.90,1.69) | 1.8% | 1.28 (1.01,1.61) | 2.0% | 1.40 (1.04,1.90) | 1.7% | 1.96 (1.33,2.88) | 1.0% | 2.62 (0.98,6.97) | 1.7% | 0.83 (0.64,1.08) |
| Rheumatoid Arthritis | 1.0% | 1.06 (0.71,1.56) | 1.5% | 1.30 (1.00,1.70) | 4.9% | 3.64 (2.89,4.58) | 1.2% | 1.47 (0.95,2.28) | 0.7% | 0.95 (0.39,2.32) | 2.9% | 1.95 (1.56,2.43) |
| Stroke (inc. TIA) | 3.9% | 1.22 (1.01,1.47) | 5.0% | 1.15 (1.00,1.32) | 4.8% | 1.11 (0.92,1.34) | 4.2% | 1.53 (1.23,1.92) | 2.2% | 1.77 (1.01,3.13) | 7.5% | 1.31 (1.15,1.48) |
|  |  |  |  |  |  |  |  |  |  |  |  |  |
| 2 or more QOF conditions | 32.0% | 1.17 (1.11,1.23) | 37.0% | 1.15 (1.10,1.19) | 44.3% | 1.33 (1.28,1.39) | 31.9% | 1.29 (1.21,1.37) | 27.5% | 1.37 (1.21,1.56) | 49.8% | 1.21 (1.17,1.25) |
| 4 or more QOF conditions | 7.3% | 1.22 (1.08,1.38) | 9.0% | 1.16 (1.06,1.28) | 12.4% | 1.48 (1.33,1.66) | 6.9% | 1.31 (1.11,1.54) | 5.1% | 2.05 (1.43,2.95) | 15.8% | 1.41 (1.30,1.53) |

**%** - prevalence in NMD patients. **PR** – prevalence ratio and 95% confidence intervals compared to non-NMD patients matched on age-sex-practice
* - Excludes non-melanoma skin cancer. ** - Includes psychosis, schizophrenia, bipolar disorder.
