# Supplementary material for: Prevalence of co-morbidity and history of recent infection in patients with neuromuscular disease: A cross-sectional analysis of United Kingdom primary care data
Source: PLoS One. 2023 Mar 1;18(3):e0282513. doi: 10.1371/journal.pone.0282513 (PMC9977045; doi:10.1371/journal.pone.0282513)
Supplement: S7 Table — (DOCX) [file pone.0282513.s009.docx]

## **Table S7:** Prevalence of other non-QOF conditions in adults with neuromuscular disease (NMD), and prevalence ratios compared to matched non-NMD patients, by type of NMD

| Condition | Charcot-Marie Tooth | | Guillain-Barré syndrome | | Inflammatory myopathies | | Muscular dystrophy | | Myotonic dystrophy (T1) | | Myasthenia Gravis | |
| --- | --- | --- | --- | --- | --- | --- | --- | --- | --- | --- | --- | --- |
|  | % | PR (95% CI) | % | PR (95% CI) | % | PR (95% CI) | % | PR (95% CI) | % | PR (95% CI) | % | PR (95% CI) |
| Cancer |  |  |  |  |  |  |  |  |  |  |  |  |
| - Non-melanoma skin | 4.3% | 0.99 (0.83,1.18) | 5.6% | 1.07 (0.94,1.21) | 6.4% | 1.36 (1.16,1.61) | 3.3% | 1.03 (0.82,1.31) | 7.7% | 3.31 (2.44,4.50) | 9.6% | 1.35 (1.21,1.51) |
| Circulatory |  |  |  |  |  |  |  |  |  |  |  |  |
| - Cardio-myopathy (any) | 0.5% | 1.38 (0.80,2.38) | 0.5% | 1.11 (0.69,1.77) | 0.8% | 2.66 (1.56,4.51) | 5.8% | 19.98 (13.22,30.20) | 8.2% | 52.37 (21.08,130.1) | 0.6% | 1.28 (0.80,2.06) |
| - Pulmonary embolism | 1.3% | 1.44 (1.02,2.03) | 2.3% | 2.00 (1.59,2.51) | 2.8% | 2.32 (1.75,3.07) | 1.6% | 1.69 (1.16,2.47) | 3.6% | 5.22 (2.94,9.28) | 3.2% | 2.20 (1.77,2.75) |
| - VTE disease (exc. PE) | 3.9% | 1.69 (1.38,2.07) | 4.6% | 1.86 (1.59,2.18) | 5.9% | 2.07 (1.72,2.50) | 3.1% | 1.42 (1.09,1.84) | 4.0% | 2.11 (1.37,3.27) | 6.1% | 1.59 (1.37,1.83) |
| Digestive |  |  |  |  |  |  |  |  |  |  |  |  |
| - Constipation | 14.9% | 1.62 (1.47,1.78) | 13.6% | 1.35 (1.24,1.46) | 15.7% | 1.35 (1.22,1.49) | 17.4% | 1.91 (1.71,2.13) | 19.6% | 2.34 (1.95,2.81) | 15.5% | 1.28 (1.18,1.39) |
| - Dysphagia | 3.4% | 1.53 (1.23,1.89) | 3.3% | 1.35 (1.13,1.62) | 7.0% | 2.57 (2.15,3.07) | 5.6% | 2.73 (2.20,3.39) | 8.6% | 5.70 (3.99,8.13) | 7.9% | 2.92 (2.54,3.37) |
| - Irritable bowel syndrome | 9.2% | 1.32 (1.16,1.49) | 8.4% | 1.17 (1.05,1.30) | 10.4% | 1.29 (1.14,1.46) | 8.7% | 1.34 (1.15,1.55) | 14.7% | 2.02 (1.64,2.50) | 8.9% | 1.15 (1.02,1.29) |
| Ear Disease |  |  |  |  |  |  |  |  |  |  |  |  |
| - Hearing Loss | 17.4% | 1.56 (1.43,1.70) | 15.6% | 1.18 (1.10,1.27) | 15.2% | 1.16 (1.06,1.28) | 12.7% | 1.22 (1.08,1.37) | 13.9% | 1.61 (1.30,2.00) | 18.8% | 1.14 (1.06,1.22) |
| Endocrine |  |  |  |  |  |  |  |  |  |  |  |  |
| - Hypothyroidism | 6.9% | 1.20 (1.05,1.37) | 8.2% | 1.15 (1.03,1.28) | 13.7% | 1.60 (1.44,1.79) | 6.9% | 1.32 (1.12,1.56) | 6.6% | 1.27 (0.94,1.71) | 15.8% | 1.78 (1.63,1.94) |
| Eye Diseases |  |  |  |  |  |  |  |  |  |  |  |  |
| - Cataract | 8.6% | 1.38 (1.23,1.55) | 10.1% | 1.20 (1.10,1.31) | 13.0% | 1.52 (1.38,1.68) | 7.2% | 1.36 (1.17,1.58) | 30.1% | 10.93 (8.36,14.30) | 17.6% | 1.45 (1.35,1.56) |
| - Glaucoma | 2.8% | 1.33 (1.06,1.68) | 3.4% | 1.26 (1.06,1.49) | 3.3% | 1.21 (0.97,1.52) | 2.5% | 1.52 (1.14,2.04) | 1.2% | 1.32 (0.63,2.78) | 5.1% | 1.29 (1.10,1.50) |
| - Macular degeneration | 1.3% | 0.91 (0.66,1.25) | 1.9% | 1.00 (0.80,1.25) | 2.5% | 1.44 (1.10,1.88) | 1.5% | 1.43 (0.98,2.08) | 1.7% | 4.61 (2.22,9.57) | 3.3% | 1.25 (1.04,1.51) |
| - Uveitis | 1.2% | 1.17 (0.82,1.69) | 1.2% | 1.01 (0.75,1.34) | 2.1% | 1.72 (1.26,2.33) | 1.2% | 1.36 (0.89,2.09) | 1.2% | 1.65 (0.75,3.62) | 1.6% | 1.26 (0.95,1.68) |
| - Visual impairment | 1.8% | 1.97 (1.45,2.69) | 1.8% | 1.39 (1.10,1.76) | 2.2% | 1.77 (1.32,2.36) | 2.2% | 2.09 (1.50,2.91) | 4.3% | 6.30 (3.71,10.70) | 2.4% | 1.48 (1.17,1.87) |
| Genitourinary |  |  |  |  |  |  |  |  |  |  |  |  |
| - Erectile dysfunction* | 20.3% | 1.53 (1.38,1.71) | 19.4% | 1.24 (1.13,1.35) | 21.0% | 1.36 (1.19,1.55) | 13.7% | 1.26 (1.08,1.46) | 18.6% | 1.49 (1.17,1.88) | 20.9% | 1.07 (0.97,1.18) |
| - Urinary Incontinence | 6.9% | 1.64 (1.42,1.90) | 6.1% | 1.29 (1.14,1.46) | 8.0% | 1.37 (1.19,1.58) | 6.9% | 1.64 (1.38,1.95) | 9.2% | 2.20 (1.69,2.86) | 10.3% | 1.69 (1.51,1.88) |
| Mental Health |  |  |  |  |  |  |  |  |  |  |  |  |
| - Anxiety disorders | 22.0% | 1.21 (1.13,1.31) | 20.8% | 1.16 (1.09,1.24) | 21.4% | 1.10 (1.01,1.19) | 18.8% | 1.09 (0.99,1.20) | 18.0% | 0.93 (0.79,1.10) | 19.1% | 1.08 (1.01,1.16) |
| -Autism/ Asperger's synd. | 0.6% | 1.76 (1.03,3.00) | 0.2% | 1.54 (0.74,3.21) | 0.3% | 1.47 (0.64,3.39) | 1.1% | 1.96 (1.23,3.12) | 1.9% | 5.44 (2.66,11.13) | 0.2% | 1.56 (0.69,3.51) |
| Musculoskeletal |  |  |  |  |  |  |  |  |  |  |  |  |
| - Collapsed vertebra | 0.7% | 2.57 (1.53,4.34) | 0.6% | 1.40 (0.92,2.13) | 1.1% | 2.02 (1.31,3.12) | 0.8% | 3.61 (1.94,6.71) | 0.5% | 2.63 (0.63,11.00) | 1.3% | 1.93 (1.37,2.71) |
| - Fracture of hip | 1.7% | 2.19 (1.60,3.00) | 1.7% | 1.41 (1.10,1.82) | 1.7% | 1.32 (0.95,1.82) | 2.5% | 3.92 (2.75,5.60) | 0.7% | 1.48 (0.52,4.20) | 1.9% | 1.20 (0.92,1.55) |
| - Fracture of wrist | 4.1% | 1.09 (0.91,1.32) | 4.2% | 1.09 (0.94,1.28) | 4.3% | 0.94 (0.78,1.14) | 3.2% | 0.89 (0.69,1.14) | 4.7% | 1.38 (0.96,1.99) | 4.5% | 1.13 (0.96,1.32) |
| - Osteoarthritis (excl spine) | 21.4% | 1.36 (1.27,1.46) | 21.1% | 1.05 (1.00,1.11) | 27.5% | 1.27 (1.20,1.35) | 14.3% | 0.97 (0.88,1.08) | 9.0% | 0.84 (0.67,1.06) | 28.6% | 1.14 (1.08,1.20) |
| - Scoliosis | 4.2% | 6.27 (4.80,8.17) | 1.2% | 1.35 (1.00,1.84) | 1.2% | 1.59 (1.06,2.36) | 7.1% | 7.94 (6.11,10.32) | 3.2% | 3.45 (2.00,5.92) | 1.3% | 1.57 (1.14,2.16) |
| - Spondylosis | 7.6% | 1.39 (1.22,1.58) | 7.2% | 1.11 (0.99,1.24) | 10.7% | 1.52 (1.35,1.72) | 6.4% | 1.34 (1.13,1.60) | 3.8% | 1.06 (0.71,1.56) | 10.8% | 1.27 (1.15,1.41) |
| Neurological |  |  |  |  |  |  |  |  |  |  |  |  |
| - Diabetic Neuropathy | 1.5% | 4.43 (2.92,6.72) | 0.9% | 2.01 (1.40,2.89) | 0.9% | 1.80 (1.12,2.89) | 0.9% | 2.53 (1.48,4.33) | 0.5% | 3.15 (0.72,13.79) | 1.1% | 1.62 (1.16,2.27) |
| - Migraine | 10.9% | 1.31 (1.17,1.47) | 9.9% | 1.27 (1.15,1.40) | 10.7% | 1.21 (1.07,1.36) | 7.4% | 0.98 (0.84,1.15) | 7.4% | 0.74 (0.57,0.98) | 10.0% | 1.24 (1.12,1.38) |
| - Multiple sclerosis | 0.7% | 3.00 (1.77,5.07) | 1.8% | 5.59 (4.00,7.82) | 0.6% | 1.54 (0.88,2.70) | 0.3% | 1.28 (0.58,2.83) | 0.2% | 0.61 (0.14,2.71) | 1.0% | 2.71 (1.80,4.08) |
| - Parkinson's disease | 0.6% | 1.51 (0.87,2.63) | 0.7% | 1.45 (0.97,2.15) | 0.7% | 1.34 (0.79,2.27) | 0.5% | 1.36 (0.70,2.64) | 0.2% | 1.31 (0.26,6.51) | 0.9% | 1.33 (0.90,1.95) |
| - Post-viral fatigue synd. | 6.1% | 1.57 (1.34,1.84) | 6.7% | 1.61 (1.42,1.83) | 10.6% | 2.37 (2.07,2.72) | 6.0% | 1.66 (1.38,2.00) | 4.6% | 1.20 (0.83,1.72) | 8.4% | 1.92 (1.70,2.18) |
| Respiratory |  |  |  |  |  |  |  |  |  |  |  |  |
| - Aspiration pneumonitis | 0.3% | 1.89 (0.89,4.01) | 0.3% | 1.43 (0.77,2.67) | 0.9% | 8.68 (4.25,17.73) | 0.8% | 6.90 (3.28,14.50) | 1.4% | 21.74 (4.73,99.99) | 0.7% | 3.45 (2.06,5.78) |
| - Sleep apnoea | 4.2% | 3.26 (2.63,4.04) | 2.2% | 1.36 (1.09,1.69) | 2.7% | 2.49 (1.87,3.31) | 4.5% | 3.46 (2.68,4.47) | 11.5% | 10.25 (6.99,15.03) | 4.2% | 2.76 (2.27,3.37) |

**%** - prevalence in NMD patients. **PR** – prevalence ratio and 95% confidence intervals compared to non-NMD patients on age-sex-practice, * - men only
