# Supplementary material for: Prevalence of co-morbidity and history of recent infection in patients with neuromuscular disease: A cross-sectional analysis of United Kingdom primary care data
Source: PLoS One. 2023 Mar 1;18(3):e0282513. doi: 10.1371/journal.pone.0282513 (PMC9977045; doi:10.1371/journal.pone.0282513)
Supplement: S8 Table — (DOCX) [file pone.0282513.s010.docx]

## **Table S8:** Occurrence of an infection in the last 12 months in all patients with neuromuscular disease (NMD), and prevalence ratios compared to matched non-NMD patients, by type of NMD

| Condition | Charcot-Marie Tooth | | Guillain-Barre syndrome | | Inflammatory myopathies | | Muscular dystrophy | | Myotonic dystrophy (T1) | | Myasthenia Gravis | |
| --- | --- | --- | --- | --- | --- | --- | --- | --- | --- | --- | --- | --- |
|  | % | PR (95% CI) | % | PR (95% CI) | % | PR (95% CI) | % | PR (95% CI) | % | PR (95% CI) | % | PR (95% CI) |
| Recorded in primary care* |  |  |  |  |  |  |  |  |  |  |  |  |
| - Any plus prescription | 15.5% | 1.34 (1.23,1.47) | 14.4% | 1.21 (1.12,1.31) | 19.0% | 1.48 (1.35,1.62) | 16.8% | 1.67 (1.51,1.85) | 20.1% | 1.80 (1.52,2.13) | 20.0% | 1.51 (1.40,1.63) |
| - Cellulitis | 2.6% | 2.13 (1.66,2.74) | 1.7% | 1.08 (0.85,1.39) | 2.7% | 1.94 (1.47,2.56) | 1.8% | 1.99 (1.40,2.82) | 2.7% | 3.23 (1.85,5.62) | 3.2% | 1.58 (1.29,1.95) |
| - Eye | 0.9% | 1.32 (0.87,2.00) | 1.0% | 1.10 (0.79,1.51) | 1.2% | 1.11 (0.75,1.65) | 1.2% | 1.52 (1.01,2.28) | 3.3% | 3.31 (2.01,5.45) | 1.2% | 1.24 (0.89,1.71) |
| - Gastro-Intestinal Tract | 0.6% | 1.06 (0.66,1.70) | 0.6% | 1.10 (0.73,1.66) | 0.9% | 1.97 (1.21,3.22) | 0.5% | 1.03 (0.57,1.84) | 1.1% | 3.12 (1.29,7.52) | 1.0% | 1.73 (1.17,2.56) |
| - Genito-Urinary | 2.8% | 1.36 (1.08,1.70) | 3.6% | 1.54 (1.29,1.84) | 3.6% | 1.38 (1.10,1.72) | 1.8% | 1.08 (0.79,1.48) | 3.5% | 1.86 (1.20,2.89) | 4.1% | 1.43 (1.20,1.72) |
| - Lower Respiratory Tract | 4.7% | 1.28 (1.08,1.53) | 5.3% | 1.22 (1.06,1.40) | 7.8% | 1.75 (1.50,2.04) | 7.1% | 2.22 (1.86,2.65) | 10.0% | 3.16 (2.35,4.24) | 9.0% | 1.64 (1.45,1.85) |
| - Mycoses - Candidiasis | 1.0% | 1.25 (0.86,1.82) | 1.0% | 1.43 (1.02,2.00) | 1.6% | 1.78 (1.25,2.55) | 1.4% | 2.14 (1.42,3.23) | 1.3% | 1.39 (0.70,2.78) | 1.5% | 1.82 (1.33,2.51) |
| - Mycoses - Other Fungal | 1.7% | 1.15 (0.85,1.54) | 1.1% | 0.76 (0.57,1.03) | 2.1% | 1.98 (1.44,2.72) | 1.8% | 1.67 (1.19,2.35) | 1.8% | 1.46 (0.80,2.67) | 2.2% | 1.57 (1.23,2.01) |
| - Skin (Other) | 5.1% | 1.37 (1.16,1.63) | 4.4% | 1.23 (1.06,1.44) | 6.1% | 1.57 (1.31,1.88) | 5.1% | 1.45 (1.20,1.76) | 6.0% | 1.51 (1.10,2.08) | 5.5% | 1.46 (1.25,1.70) |
| - Upper Respiratory Tract | 7.8% | 1.26 (1.10,1.44) | 6.1% | 1.11 (0.98,1.27) | 7.3% | 1.17 (1.00,1.36) | 8.4% | 1.37 (1.19,1.59) | 6.3% | 0.91 (0.68,1.22) | 7.3% | 1.36 (1.19,1.55) |
|  |  |  |  |  |  |  |  |  |  |  |  |  |
| Hospitalisations† |  |  |  |  |  |  |  |  |  |  |  |  |
| - Any | 4.6% | 2.25 (1.81,2.79) | 4.1% | 1.35 (1.13,1.60) | 5.7% | 1.91 (1.56,2.34) | 5.3% | 2.49 (1.96,3.17) | 6.0% | 2.74 (1.85,4.08) | 6.7% | 1.85 (1.57,2.17) |
| - Gastro-Intestinal Tract | 0.7% | 1.88 (1.10,3.20) | 0.7% | 1.44 (0.93,2.24) | 1.2% | 2.36 (1.48,3.78) | 0.8% | 1.75 (0.97,3.15) | 1.4% | 3.20 (1.32,7.71) | 0.9% | 1.83 (1.17,2.86) |
| - Lower Respiratory Tract | 1.1% | 1.51 (0.99,2.30) | 1.4% | 1.15 (0.86,1.54) | 2.4% | 2.23 (1.61,3.10) | 2.9% | 4.07 (2.82,5.88) | 2.9% | 8.25 (3.73,18.23) | 3.0% | 1.78 (1.39,2.27) |
| - Sepsis | 0.5% | 1.71 (0.88,3.29) | 0.6% | 1.79 (1.08,2.98) | 0.8% | 2.16 (1.18,3.95) | 0.6% | 2.36 (1.15,4.82) | 0.2% | 0.78 (0.09,6.68) | 1.3% | 3.37 (2.19,5.20) |

**%** - prevalence in NMD patients. **PR** – prevalence ratio and 95% confidence intervals compared to non-NMD patients matched on age-sex-practice

* - Analysis based on 22,946 NMD patients who were actively registered throughout 2018 (and 87,959 corresponding age-sex-practice matched non-NMD patients)

† - Analysis based on 19,012 NMD patients who were eligible to be linked to English Hospital Episodes Statistics (HES) data (and corresponding 74,831 age-sex-practice matched non-NMD patients)
