# Supplementary material for: Prevalence of co-morbidity and history of recent infection in patients with neuromuscular disease: A cross-sectional analysis of United Kingdom primary care data
Source: PLoS One. 2023 Mar 1;18(3):e0282513. doi: 10.1371/journal.pone.0282513 (PMC9977045; doi:10.1371/journal.pone.0282513)
Supplement: Attachment — Submitted filename: Response to reviewers.docx [file pone.0282513.s011.docx]

Reviewers' comments:

Reviewer's Responses to Questions

5. Review Comments to the Author

Please use the space provided to explain your answers to the questions above. You may also include additional comments for the author, including concerns about dual publication, research ethics, or publication ethics. (Please upload your review as an attachment if it exceeds 20,000 characters)

Reviewer #1: The authors conducted a matched case-control study to compare the prevalence of chronic health disorders between neuromuscular disorder (NMD) patients with the age-, sex- and GP practice-matched controls using a large UK primary database. As expected, NMD patients had far more comorbidities and infection than the controls.

The manuscript is overall informative. However, there are a couple of methodological issues the authors might wish to address in their subsequent submission.

We thank the reviewer for their positive summary of our paper. However, we would like to clarify that the study is strictly not a “case-control” study but better described as a “cross-sectional analysis” (as we have indicated in the title).

On reflection we think the use of the word “controls” here was misleading and our comparator group is better described as “Non-NMD” or “Patients without a NMD”.

As a result, we have clarified the description of these patients in the study throughout the manuscript.

1. Study rationales
The authors indicated, in the Introduction section that a study in NMD elderly patients might be of interest as the elderly with NMD are at an increased risk of falls and fracture and previous studies “were based primarily on younger patients”. However, their studies also included young patients with almost 40% younger than 50 years old. The authors thus might wish to make the study rationales more appealing. For instance, please clarify why this study was needed given previous findings.

We agree that our sentence was not definitive enough in making the case for our study/analysis. The existing literature for many NMD’s such as Duchene muscular dystrophy is almost exclusively based on younger patients. Even though the reviewer is correct to say about 40% of our cohort is less than 50 years old, that still leaves almost 15,000 total patients with a NMD ages 50 and over. No other study has reported on older patients on that scale before.

We have amended the end of paragraph 2 to now say

“Earlier studies of some NMDs, such as Duchene muscular dystrophy have been based almost exclusively primarily on younger patients, who may be less representative of the overall disease burden in the wider population as the associated life expectancy with the condition has increased over time[10]. As NMD patients are already at a greater risk of falls and fractures from a loss of muscle power over time[1], this risk may become more relevant to an ageing patient group. However, there is an absence of large-scale descriptive studies of older patients with NMD. Better recognition of older patients with a NMD is important, since they are likely to be frequently hospitalised, so better coordinated care might prevent some admissions such as fractures and infections[11].”

2. Clusters of comorbidities might be more informative and more clinically relevant than a set of specific chronic diseases
The authors might find the identification of specific clusters of comorbidities in NMD patients more clinically relevant than a set of specific diseases given the inter-correlation between the diseases. Statistical approaches, such as a latent class analysis are widely available for such an analysis.
The authors might wish to discuss why they did consider identification of a set of specific diseases more appropriate in the study than that of the cluster of comorbidities, which has been found to be clinically relevant and statistically robust.

The reviewer is right to point out that there will be clusters of diseases/conditions specific to different NMDs. However, our objective was to describe individual diseases or conditions compared to a general population sample (“controls”). We would argue this is a necessary first step in any comparison, is more directly informative, and ultimately more clinically relevant.

We agree that multi-morbidity is an important issue and did already include a count of diseases in Table 2. When we did explore specific combinations of the diseases in Table 2 by NMD condition it tended to show what one might expect, with the most common diseases (asthma, depression, hypertension etc) also the most likely to be in cluster. Exploring any cluster differences between NMD condition is not straightforward because of the underlying differences in average age for some of the NMD conditions.

Therefore, we are not convinced that a latent class analysis would necessarily be helpful, primarily because you would need to pre-specify the number of latent classes (or clusters). We think an agglomerative hierarchical clustering analysis would be more appropriate. However, this would be a complex undertaking, and beyond the scope of the current analysis which is based on comparisons between patients with and without NMD.

3. Validation of NMD diagnosis
The study did not validate the diagnosis of NMD, and the authors have acknowledged it as a study limitation. It would be more convincing if they would discuss further on how the mis-classification bias would have affected the findings (i.e., the direction and magnitude of the mis-classification bias).

As the reviewer correctly notes we did not attempt to validate the diagnoses, as these diagnoses are more reliably made in a specialist care setting and then transferred onto the GP system. We do already note in the discussion (lines 317-9) that – “while it is possible that some of the patients may have been mis-diagnosed or mis-classified, that would lead to our analysis underestimating the elevated associations we described”.

4. NMD patients with multiple conditions
There was “a small number of patients had multiple conditions” of NMD (line 102-103). Unfortunately, findings of this group are mentioned at all in the manuscript though they might represent the group of NMD patients with the highest risk of comorbidities. Please add their findings and discuss them.

We apologise for the oversight in not providing a number here – it was only provided in the footnote to Table 1. A total of 183 (0.8%) of the patients with a NMD in our study had diagnoses resulting in them being classified in more than one of our groups (Charcot-Marie Tooth, Guillain-Barré syndrome, inflammatory myopathies, muscular dystrophy, myotonic dystrophy type 1, myasthenia gravis). We suspect the presence of multiple diagnoses may reflect diagnostic uncertainty in many instances as the dates of diagnoses are often recorded close in time. We already observed (line 384) a few instances where Guillain-Barré syndrome and multiple sclerosis diagnoses had the same date for example, with the implication being that only one may indeed be correct.

As the number of these patients is so small, and the reason surrounding the multiple diagnoses unclear, we do not think presenting this group of patients would be a meaningful addition to the analysis. Nor would excluding them (due to any uncertainty over the diagnosis) make much of an impact. However, we have added the following line to the results

“A small number of patients (n=183) were classified into multiple NMD categories and appear in the analysis for each group.”

5. Controls
The controls were selected from those without a history of NMD in the same GP practice registered > 90 days. The selection of controls should be described in more details. For instance, please clarify whether the controls were mutually exclusive (i.e., the controls were automatically selected without replacement and it is not possible that one person served as a control for more than one case), and whether any mathematic algorithm was conducted to automatically and systematically select the controls.

The controls were randomly selected without replacement, so all 95,295 “controls” are unique patients. An algorithm written within SAS selects the patients randomly. We now say

“A total of 95,295 patients without a NMD were randomly selected without replacement.”

6. Minor:
a. Table 1: please add percentages (%) into the “Number of controls” column for better comparison.

We have added these now (though these are virtually identical because of the matching)

b. Table 1: please add details of the NMD patients with multiple NMD conditions.

The number was already indicated in the footnote, but we have added to the results now. As discussed above, we don’t believe any further description of them is needed.

c. The authors might wish to re-arrange the Discussion section as we don’t usually discuss the “Limitations” before the “Selected and Key Findings”.

We have amended discussion now by (i) including a new paragraph highlighting the strengths of the study before we discuss the limitations, (ii) removing these sub-headings which we think in retrospect were misleading in places.

Reviewer #2: Thank you for your interesting manuscript. However, I have some comments for the better one.

1. The flow chart could be used for the presentation of study cohort and matched controls in the methodology.

A new supplementary chart (Figure S1) has been added

2. Concerning the discussion, the content could be widely discussed by citing relevant references, based on your findings, rather than the presentation of selected and key findings as they were reported in the section of results.

As noted above we felt these sub-headings were potentially mis-leading and have removed them. We have some other minor edits to the discussion, but overall we think our discussion gives a broad and fair overview of relevant references for each main neuromuscular disease. As outlined in the introduction, there are few extensive studies to directly compare with, so often the evidence is specific to a particular type of neuromuscular disease or condition.

3. In addition, the limitation and strength of the study could be described by following the discussion.

We have inserted a new paragraph on the strengths of the study first now.

“The main strength of our study is the size, containing over 20,000 patients with recorded NMD from a nationwide sample of general practices in the UK. The CPRD database, at the time of our study date (January 1st 2019), contained approximately 12 million registered patients representing almost 20% of the UK population[9]. So, the results are likely to be generalisable in terms of what is being recorded on primary care medical records across the UK. However, there are several limitations to our analyses.”

4. Could you add the generalizability of the study please?

We have added a sentence in the new second paragraph in the discussion (see point 3).
