# Supplementary material for: Prevalence of co-morbidity and history of recent infection in patients with neuromuscular disease: A cross-sectional analysis of United Kingdom primary care data
Source: PLoS One. 2023 Mar 1;18(3):e0282513. doi: 10.1371/journal.pone.0282513 (PMC9977045; doi:10.1371/journal.pone.0282513)
Supplement: Peer review history

# Peer review: Decision Letter 0

**DOI:** 10.1371/journal.pone.0282513.r001

**Authors:** Robert Daniel Blank

23 Nov 2022

PONE-D-22-22002Prevalence of co-morbidity and history of recent infection in patients with neuromuscular disorders: a cross-sectional analysis of United Kingdom primary care dataPLOS ONE

Dear Dr. Carey,

Thank you for submitting your manuscript to PLOS ONE. After careful consideration, we feel that it has merit but does not fully meet PLOS ONE’s publication criteria as it currently stands. Therefore, we invite you to submit a revised version of the manuscript that addresses the points raised during the review process. Each of the reviewers has provided useful guidance for improving the MS.  The flow chart suggested by reviewer 2 would be particularly welcome.

Please submit your revised manuscript by Jan 07 2023 11:59PM. If you will need more time than this to complete your revisions, please reply to this message or contact the journal office at plosone@plos.org. When you're ready to submit your revision, log on to https://www.editorialmanager.com/pone/ and select the 'Submissions Needing Revision' folder to locate your manuscript file.

Please include the following items when submitting your revised manuscript:

- A rebuttal letter that responds to each point raised by the academic editor and reviewer(s). You should upload this letter as a separate file labeled 'Response to Reviewers'.
- A marked-up copy of your manuscript that highlights changes made to the original version. You should upload this as a separate file labeled 'Revised Manuscript with Track Changes'.
- An unmarked version of your revised paper without tracked changes. You should upload this as a separate file labeled 'Manuscript'.

If you would like to make changes to your financial disclosure, please include your updated statement in your cover letter. Guidelines for resubmitting your figure files are available below the reviewer comments at the end of this letter.

If applicable, we recommend that you deposit your laboratory protocols in protocols.io to enhance the reproducibility of your results. Protocols.io assigns your protocol its own identifier (DOI) so that it can be cited independently in the future. For instructions see: https://journals.plos.org/plosone/s/submission-guidelines#loc-laboratory-protocols. Additionally, PLOS ONE offers an option for publishing peer-reviewed Lab Protocol articles, which describe protocols hosted on protocols.io. Read more information on sharing protocols at https://plos.org/protocols?utm_medium=editorial-email&utm_source=authorletters&utm_campaign=protocols.

We look forward to receiving your revised manuscript.

Kind regards,

Robert Daniel Blank, MD, PhD

Academic Editor

PLOS ONE

Journal requirements:

When submitting your revision, we need you to address these additional requirements.

1. Please ensure that your manuscript meets PLOS ONE's style requirements, including those for file naming. The PLOS ONE style templates can be found at

https://journals.plos.org/plosone/s/file?id=wjVg/PLOSOne_formatting_sample_main_body.pdf and

https://journals.plos.org/plosone/s/fileid=ba62/PLOSOne_formatting_sample_title_authors_affiliations.pdf.

2. Please note that in order to use the direct billing option the corresponding author must be affiliated with the chosen institute. Please either amend your manuscript to change the affiliation or corresponding author, or email us at plosone@plos.org with a request to remove this option.

3. In your Data Availability statement, you have not specified where the minimal data set underlying the results described in your manuscript can be found. PLOS defines a study's minimal data set as the underlying data used to reach the conclusions drawn in the manuscript and any additional data required to replicate the reported study findings in their entirety. All PLOS journals require that the minimal data set be made fully available. For more information about our data policy, please see http://journals.plos.org/plosone/s/data-availability.

Upon re-submitting your revised manuscript, please upload your study’s minimal underlying data set as either Supporting Information files or to a stable, public repository and include the relevant URLs, DOIs, or accession numbers within your revised cover letter. For a list of acceptable repositories, please see http://journals.plos.org/plosone/s/data-availability#loc-recommended-repositories. Any potentially identifying patient information must be fully anonymized.

Important: If there are ethical or legal restrictions to sharing your data publicly, please explain these restrictions in detail. Please see our guidelines for more information on what we consider unacceptable restrictions to publicly sharing data: http://journals.plos.org/plosone/s/data-availability#loc-unacceptable-data-access-restrictions. Note that it is not acceptable for the authors to be the sole named individuals responsible for ensuring data access.

We will update your Data Availability statement to reflect the information you provide in your cover letter.

4. We note that you have indicated that data from this study are available upon request. PLOS only allows data to be available upon request if there are legal or ethical restrictions on sharing data publicly. For more information on unacceptable data access restrictions, please see http://journals.plos.org/plosone/s/data-availability#loc-unacceptable-data-access-restrictions.

In your revised cover letter, please address the following prompts:

a) If there are ethical or legal restrictions on sharing a de-identified data set, please explain them in detail (e.g., data contain potentially sensitive information, data are owned by a third-party organization, etc.) and who has imposed them (e.g., an ethics committee). Please also provide contact information for a data access committee, ethics committee, or other institutional body to which data requests may be sent.

b) If there are no restrictions, please upload the minimal anonymized data set necessary to replicate your study findings as either Supporting Information files or to a stable, public repository and provide us with the relevant URLs, DOIs, or accession numbers. For a list of acceptable repositories, please see http://journals.plos.org/plosone/s/data-availability#loc-recommended-repositories.

We will update your Data Availability statement on your behalf to reflect the information you provide.

Please review your reference list to ensure that it is complete and correct. If you have cited papers that have been retracted, please include the rationale for doing so in the manuscript text, or remove these references and replace them with relevant current references. Any changes to the reference list should be mentioned in the rebuttal letter that accompanies your revised manuscript. If you need to cite a retracted article, indicate the article’s retracted status in the References list and also include a citation and full reference for the retraction notice.

[Note: HTML markup is below. Please do not edit.]

Reviewers' comments:

Reviewer's Responses to Questions

**Comments to the Author**

1. Is the manuscript technically sound, and do the data support the conclusions?

The manuscript must describe a technically sound piece of scientific research with data that supports the conclusions. Experiments must have been conducted rigorously, with appropriate controls, replication, and sample sizes. The conclusions must be drawn appropriately based on the data presented. 

Reviewer #1: Yes

Reviewer #2: Yes

**********

2. Has the statistical analysis been performed appropriately and rigorously? 

Reviewer #1: Yes

Reviewer #2: Yes

**********

3. Have the authors made all data underlying the findings in their manuscript fully available?

The PLOS Data policy requires authors to make all data underlying the findings described in their manuscript fully available without restriction, with rare exception (please refer to the Data Availability Statement in the manuscript PDF file). The data should be provided as part of the manuscript or its supporting information, or deposited to a public repository. For example, in addition to summary statistics, the data points behind means, medians and variance measures should be available. If there are restrictions on publicly sharing data—e.g. participant privacy or use of data from a third party—those must be specified.

Reviewer #1: Yes

Reviewer #2: Yes

**********

4. Is the manuscript presented in an intelligible fashion and written in standard English?

PLOS ONE does not copyedit accepted manuscripts, so the language in submitted articles must be clear, correct, and unambiguous. Any typographical or grammatical errors should be corrected at revision, so please note any specific errors here.

Reviewer #1: Yes

Reviewer #2: Yes

**********

5. Review Comments to the Author

Please use the space provided to explain your answers to the questions above. You may also include additional comments for the author, including concerns about dual publication, research ethics, or publication ethics. (Please upload your review as an attachment if it exceeds 20,000 characters)

Reviewer #1: The authors conducted a matched case-control study to compare the prevalence of chronic health disorders between neuromuscular disorder (NMD) patients with the age-, sex- and GP practice-matched controls using a large UK primary database. As expected, NMD patients had far more comorbidities and infection than the controls.

The manuscript is overall informative. However, there are a couple of methodological issues the authors might wish to address in their subsequent submission.

1. Study rationales

The authors indicated, in the Introduction section that a study in NMD elderly patients might be of interest as the elderly with NMD are at an increased risk of falls and fracture and previous studies “were based primarily on younger patients”. However, their studies also included young patients with almost 40% younger than 50 years old. The authors thus might wish to make the study rationales more appealing. For instance, please clarify why this study was needed given previous findings.

2. Clusters of comorbidities might be more informative and more clinically relevant than a set of specific chronic diseases

The authors might find the identification of specific clusters of comorbidities in NMD patients more clinically relevant than a set of specific diseases given the inter-correlation between the diseases. Statistical approaches, such as a latent class analysis are widely available for such an analysis.

The authors might wish to discuss why they did consider identification of a set of specific diseases more appropriate in the study than that of the cluster of comorbidities, which has been found to be clinically relevant and statistically robust.

3. Validation of NMD diagnosis

The study did not validate the diagnosis of NMD, and the authors have acknowledged it as a study limitation. It would be more convincing if they would discuss further on how the mis-classification bias would have affected the findings (i.e., the direction and magnitude of the mis-classification bias).

4. NMD patients with multiple conditions

There was “a small number of patients had multiple conditions” of NMD (line 102-103). Unfortunately, findings of this group are mentioned at all in the manuscript though they might represent the group of NMD patients with the highest risk of comorbidities. Please add their findings and discuss them.

5. Controls

The controls were selected from those without a history of NMD in the same GP practice registered > 90 days. The selection of controls should be described in more details. For instance, please clarify whether the controls were mutually exclusive (i.e., the controls were automatically selected without replacement and it is not possible that one person served as a control for more than one case), and whether any mathematic algorithm was conducted to automatically and systematically select the controls.

6. Minor:

a. Table 1: please add percentages (%) into the “Number of controls” column for better comparison.

b. Table 1: please add details of the NMD patients with multiple NMD conditions.

c. The authors might wish to re-arrange the Discussion section as we don’t usually discuss the “Limitations” before the “Selected and Key Findings”.

Reviewer #2: Thank you for your interesting manuscript. However, I have some comments for the better one.

1. The flow chart could be used for the presentation of study cohort and matched controls in the methodology.

2. Concerning the discussion, the content could be widely discussed by citing relevant references, based on your findings, rather than the presentation of selected and key findings as they were reported in the section of results.

3. In addition, the limitation and strength of the study could be described by following the discussion.

4. Could you add the generalizability of the study please?

**********

6. PLOS authors have the option to publish the peer review history of their article (what does this mean?). If published, this will include your full peer review and any attached files.

If you choose “no”, your identity will remain anonymous but your review may still be made public.

**Do you want your identity to be public for this peer review?** For information about this choice, including consent withdrawal, please see our Privacy Policy.

Reviewer #1: **Yes: **Thach Tran

Reviewer #2: No

**********

[NOTE: If reviewer comments were submitted as an attachment file, they will be attached to this email and accessible via the submission site. Please log into your account, locate the manuscript record, and check for the action link "View Attachments". If this link does not appear, there are no attachment files.]

While revising your submission, please upload your figure files to the Preflight Analysis and Conversion Engine (PACE) digital diagnostic tool, https://pacev2.apexcovantage.com/. PACE helps ensure that figures meet PLOS requirements. To use PACE, you must first register as a user. Registration is free. Then, login and navigate to the UPLOAD tab, where you will find detailed instructions on how to use the tool. If you encounter any issues or have any questions when using PACE, please email PLOS at figures@plos.org. Please note that Supporting Information files do not need this step.

# Author comment: Author response to Decision Letter 0

**DOI:** 10.1371/journal.pone.0282513.r002

12 Jan 2023

Reviewers' comments:

Reviewer's Responses to Questions

________________________________________

5. Review Comments to the Author

Please use the space provided to explain your answers to the questions above. You may also include additional comments for the author, including concerns about dual publication, research ethics, or publication ethics. (Please upload your review as an attachment if it exceeds 20,000 characters)

Reviewer #1: The authors conducted a matched case-control study to compare the prevalence of chronic health disorders between neuromuscular disorder (NMD) patients with the age-, sex- and GP practice-matched controls using a large UK primary database. As expected, NMD patients had far more comorbidities and infection than the controls.

The manuscript is overall informative. However, there are a couple of methodological issues the authors might wish to address in their subsequent submission.

> We thank the reviewer for their positive summary of our paper. However, we would like to clarify that the study is strictly not a “case-control” study but better described as a “cross-sectional analysis” (as we have indicated in the title). 

On reflection we think the use of the word “controls” here was misleading and our comparator group is better described as “Non-NMD” or “Patients without a NMD”.

As a result, we have clarified the description of these patients in the study throughout the manuscript.

1. Study rationales

The authors indicated, in the Introduction section that a study in NMD elderly patients might be of interest as the elderly with NMD are at an increased risk of falls and fracture and previous studies “were based primarily on younger patients”. However, their studies also included young patients with almost 40% younger than 50 years old. The authors thus might wish to make the study rationales more appealing. For instance, please clarify why this study was needed given previous findings.

> We agree that our sentence was not definitive enough in making the case for our study/analysis. The existing literature for many NMD’s such as Duchene muscular dystrophy is almost exclusively based on younger patients. Even though the reviewer is correct to say about 40% of our cohort is less than 50 years old, that still leaves almost 15,000 total patients with a NMD ages 50 and over. No other study has reported on older patients on that scale before.

We have amended the end of paragraph 2 to now say 

“Earlier studies of some NMDs, such as Duchene muscular dystrophy have been based almost exclusively primarily on younger patients, who may be less representative of the overall disease burden in the wider population as the associated life expectancy with the condition has increased over time[10]. As NMD patients are already at a greater risk of falls and fractures from a loss of muscle power over time[1], this risk may become more relevant to an ageing patient group. However, there is an absence of large-scale descriptive studies of older patients with NMD. Better recognition of older patients with a NMD is important, since they are likely to be frequently hospitalised, so better coordinated care might prevent some admissions such as fractures and infections[11].”

2. Clusters of comorbidities might be more informative and more clinically relevant than a set of specific chronic diseases

The authors might find the identification of specific clusters of comorbidities in NMD patients more clinically relevant than a set of specific diseases given the inter-correlation between the diseases. Statistical approaches, such as a latent class analysis are widely available for such an analysis.

The authors might wish to discuss why they did consider identification of a set of specific diseases more appropriate in the study than that of the cluster of comorbidities, which has been found to be clinically relevant and statistically robust.

> The reviewer is right to point out that there will be clusters of diseases/conditions specific to different NMDs. However, our objective was to describe individual diseases or conditions compared to a general population sample (“controls”). We would argue this is a necessary first step in any comparison, is more directly informative, and ultimately more clinically relevant.

We agree that multi-morbidity is an important issue and did already include a count of diseases in Table 2. When we did explore specific combinations of the diseases in Table 2 by NMD condition it tended to show what one might expect, with the most common diseases (asthma, depression, hypertension etc) also the most likely to be in cluster. Exploring any cluster differences between NMD condition is not straightforward because of the underlying differences in average age for some of the NMD conditions. 

Therefore, we are not convinced that a latent class analysis would necessarily be helpful, primarily because you would need to pre-specify the number of latent classes (or clusters). We think an agglomerative hierarchical clustering analysis would be more appropriate. However, this would be a complex undertaking, and beyond the scope of the current analysis which is based on comparisons between patients with and without NMD.

3. Validation of NMD diagnosis

The study did not validate the diagnosis of NMD, and the authors have acknowledged it as a study limitation. It would be more convincing if they would discuss further on how the mis-classification bias would have affected the findings (i.e., the direction and magnitude of the mis-classification bias).

>As the reviewer correctly notes we did not attempt to validate the diagnoses, as these diagnoses are more reliably made in a specialist care setting and then transferred onto the GP system. We do already note in the discussion (lines 317-9) that – “while it is possible that some of the patients may have been mis-diagnosed or mis-classified, that would lead to our analysis underestimating the elevated associations we described”. 

4. NMD patients with multiple conditions

There was “a small number of patients had multiple conditions” of NMD (line 102-103). Unfortunately, findings of this group are mentioned at all in the manuscript though they might represent the group of NMD patients with the highest risk of comorbidities. Please add their findings and discuss them.

> We apologise for the oversight in not providing a number here – it was only provided in the footnote to Table 1. A total of 183 (0.8%) of the patients with a NMD in our study had diagnoses resulting in them being classified in more than one of our groups (Charcot-Marie Tooth, Guillain-Barré syndrome, inflammatory myopathies, muscular dystrophy, myotonic dystrophy type 1, myasthenia gravis). We suspect the presence of multiple diagnoses may reflect diagnostic uncertainty in many instances as the dates of diagnoses are often recorded close in time. We already observed (line 384) a few instances where Guillain-Barré syndrome and multiple sclerosis diagnoses had the same date for example, with the implication being that only one may indeed be correct. 

As the number of these patients is so small, and the reason surrounding the multiple diagnoses unclear, we do not think presenting this group of patients would be a meaningful addition to the analysis. Nor would excluding them (due to any uncertainty over the diagnosis) make much of an impact. However, we have added the following line to the results

“A small number of patients (n=183) were classified into multiple NMD categories and appear in the analysis for each group.”

5. Controls

The controls were selected from those without a history of NMD in the same GP practice registered > 90 days. The selection of controls should be described in more details. For instance, please clarify whether the controls were mutually exclusive (i.e., the controls were automatically selected without replacement and it is not possible that one person served as a control for more than one case), and whether any mathematic algorithm was conducted to automatically and systematically select the controls.

>The controls were randomly selected without replacement, so all 95,295 “controls” are unique patients. An algorithm written within SAS selects the patients randomly. We now say

“A total of 95,295 patients without a NMD were randomly selected without replacement.”

6. Minor:

a. Table 1: please add percentages (%) into the “Number of controls” column for better comparison.

>We have added these now (though these are virtually identical because of the matching)

b. Table 1: please add details of the NMD patients with multiple NMD conditions.

>The number was already indicated in the footnote, but we have added to the results now. As discussed above, we don’t believe any further description of them is needed. 

c. The authors might wish to re-arrange the Discussion section as we don’t usually discuss the “Limitations” before the “Selected and Key Findings”.

>We have amended discussion now by (i) including a new paragraph highlighting the strengths of the study before we discuss the limitations, (ii) removing these sub-headings which we think in retrospect were misleading in places.

Reviewer #2: Thank you for your interesting manuscript. However, I have some comments for the better one.

1. The flow chart could be used for the presentation of study cohort and matched controls in the methodology.

>A new supplementary chart (Figure S1) has been added

2. Concerning the discussion, the content could be widely discussed by citing relevant references, based on your findings, rather than the presentation of selected and key findings as they were reported in the section of results.

>As noted above we felt these sub-headings were potentially mis-leading and have removed them. We have some other minor edits to the discussion, but overall we think our discussion gives a broad and fair overview of relevant references for each main neuromuscular disease. As outlined in the introduction, there are few extensive studies to directly compare with, so often the evidence is specific to a particular type of neuromuscular disease or condition. 

3. In addition, the limitation and strength of the study could be described by following the discussion.

>We have inserted a new paragraph on the strengths of the study first now.

“The main strength of our study is the size, containing over 20,000 patients with recorded NMD from a nationwide sample of general practices in the UK. The CPRD database, at the time of our study date (January 1st 2019), contained approximately 12 million registered patients representing almost 20% of the UK population[9]. So, the results are likely to be generalisable in terms of what is being recorded on primary care medical records across the UK. However, there are several limitations to our analyses.”

4. Could you add the generalizability of the study please?

>We have added a sentence in the new second paragraph in the discussion (see point 3).

Attachment Submitted filename: Response to reviewers.docx Click here for additional data file.

# Peer review: Decision Letter 1

**DOI:** 10.1371/journal.pone.0282513.r003

**Authors:** Robert Daniel Blank

17 Feb 2023

Prevalence of co-morbidity and history of recent infection in patients with neuromuscular disease: a cross-sectional analysis of United Kingdom primary care data

PONE-D-22-22002R1

Dear Dr. Carey,

We’re pleased to inform you that your manuscript has been judged scientifically suitable for publication and will be formally accepted for publication once it meets all outstanding technical requirements.

Within one week, you’ll receive an e-mail detailing the required amendments. When these have been addressed, you’ll receive a formal acceptance letter and your manuscript will be scheduled for publication.

An invoice for payment will follow shortly after the formal acceptance. To ensure an efficient process, please log into Editorial Manager at http://www.editorialmanager.com/pone/, click the 'Update My Information' link at the top of the page, and double check that your user information is up-to-date. If you have any billing related questions, please contact our Author Billing department directly at authorbilling@plos.org.

If your institution or institutions have a press office, please notify them about your upcoming paper to help maximize its impact. If they’ll be preparing press materials, please inform our press team as soon as possible -- no later than 48 hours after receiving the formal acceptance. Your manuscript will remain under strict press embargo until 2 pm Eastern Time on the date of publication. For more information, please contact onepress@plos.org.

Kind regards,

Robert Daniel Blank, MD, PhD

Academic Editor

PLOS ONE

Additional Editor Comments (optional):

Reviewers' comments:

Reviewer's Responses to Questions

**Comments to the Author**

1. If the authors have adequately addressed your comments raised in a previous round of review and you feel that this manuscript is now acceptable for publication, you may indicate that here to bypass the “Comments to the Author” section, enter your conflict of interest statement in the “Confidential to Editor” section, and submit your "Accept" recommendation.

Reviewer #1: All comments have been addressed

Reviewer #2: All comments have been addressed

**********

2. Is the manuscript technically sound, and do the data support the conclusions?

The manuscript must describe a technically sound piece of scientific research with data that supports the conclusions. Experiments must have been conducted rigorously, with appropriate controls, replication, and sample sizes. The conclusions must be drawn appropriately based on the data presented. 

Reviewer #1: Yes

Reviewer #2: Yes

**********

3. Has the statistical analysis been performed appropriately and rigorously? 

Reviewer #1: Yes

Reviewer #2: Yes

**********

4. Have the authors made all data underlying the findings in their manuscript fully available?

The PLOS Data policy requires authors to make all data underlying the findings described in their manuscript fully available without restriction, with rare exception (please refer to the Data Availability Statement in the manuscript PDF file). The data should be provided as part of the manuscript or its supporting information, or deposited to a public repository. For example, in addition to summary statistics, the data points behind means, medians and variance measures should be available. If there are restrictions on publicly sharing data—e.g. participant privacy or use of data from a third party—those must be specified.

Reviewer #1: Yes

Reviewer #2: Yes

**********

5. Is the manuscript presented in an intelligible fashion and written in standard English?

PLOS ONE does not copyedit accepted manuscripts, so the language in submitted articles must be clear, correct, and unambiguous. Any typographical or grammatical errors should be corrected at revision, so please note any specific errors here.

Reviewer #1: Yes

Reviewer #2: Yes

**********

6. Review Comments to the Author

Please use the space provided to explain your answers to the questions above. You may also include additional comments for the author, including concerns about dual publication, research ethics, or publication ethics. (Please upload your review as an attachment if it exceeds 20,000 characters)

Reviewer #1: I would like to thank the authors for their effort to address my concerns. I find the authors' responses are all favourable.

Reviewer #2: I have no more suggestions for this manuscript as you already have addressed all points I commneted.

**********

7. PLOS authors have the option to publish the peer review history of their article (what does this mean?). If published, this will include your full peer review and any attached files.

If you choose “no”, your identity will remain anonymous but your review may still be made public.

**Do you want your identity to be public for this peer review?** For information about this choice, including consent withdrawal, please see our Privacy Policy.

Reviewer #1: No

Reviewer #2: No

**********

# Editor report: Acceptance letter

**DOI:** 10.1371/journal.pone.0282513.r004

**Authors:** Robert Daniel Blank

21 Feb 2023

PONE-D-22-22002R1 

Prevalence of co-morbidity and history of recent infection in patients with neuromuscular disease: a cross-sectional analysis of United Kingdom primary care data 

Dear Dr. Carey:

I'm pleased to inform you that your manuscript has been deemed suitable for publication in PLOS ONE. Congratulations! Your manuscript is now with our production department. 

If your institution or institutions have a press office, please let them know about your upcoming paper now to help maximize its impact. If they'll be preparing press materials, please inform our press team within the next 48 hours. Your manuscript will remain under strict press embargo until 2 pm Eastern Time on the date of publication. For more information please contact onepress@plos.org.

If we can help with anything else, please email us at plosone@plos.org. 

Thank you for submitting your work to PLOS ONE and supporting open access. 

Kind regards, 

PLOS ONE Editorial Office Staff

on behalf of

Professor Robert Daniel Blank 

Academic Editor

PLOS ONE
